# Chrysin as a Multifunctional Therapeutic Flavonoid: Emerging Insights in Pathogenesis Management: A Narrative Review

**DOI:** 10.3390/ijms27010072

**Published:** 2025-12-21

**Authors:** Arshad Husain Rahmani, Amjad Ali Khan

**Affiliations:** 1 Department of Medical Laboratories, College of Applied Medical Sciences, Qassim University, Buraydah 51452, Saudi Arabia; 2 Department of Basic Health Sciences, College of Applied Medical Sciences, Qassim University, Buraydah 51452, Saudi Arabia; akhan@qu.edu.sa

**Keywords:** chrysin, pathogenesis, inflammation and oxidative stress, cancer therapy

## Abstract

Chrysin, a vital flavonoid found in fruits, vegetables, honey, and propolis, plays a significant role in the management of various pathogenesis. Its ability to reduce oxidative stress and mitigate inflammation is a reassuring factor in disease management. In addition, its role in various cancers has been demonstrated and it modulates cell signaling pathways, including inflammation, angiogenesis, apoptosis, autophagy, and the cell cycle. The literature was collected using search engines such as Google, Google Scholar, PubMed, and Scopus. Keywords included chrysin sources, antioxidant and anti-inflammatory activity, cardioprotective and hepatoprotective effects, as well as anti-diabetic, neuroprotective, anti-cancer, antimicrobial, and bone-protective roles. Research and review articles, along with relevant clinical trials published in English, were included. This narrative review summarizes the therapeutic potential of chrysin in the management of chronic diseases. Additionally, combination therapies of chrysin with other drugs/natural compounds provide synergistic benefits, leading to increased efficacy and lower toxicity. Despite its promising pharmacological activities, the clinical utility of chrysin remains limited due to its poor bioavailability, low solubility, limited permeability, and rapid metabolism. Overcoming these challenges will require the development of advanced formulations, mechanistic studies, and well-designed clinical trials to fully exploit chrysin’s potential role in disease management.

## 1. Introduction

Natural compounds and their active constituents play a substantial role in both the prevention and treatment of numerous diseases [1,2]. Their effectiveness in disease prevention is primarily attributed to their strong antioxidant and anti-inflammatory properties, which help to counteract oxidative stress and reduce inflammation [3]. These compounds are often not only effective but also inexpensive and generally regarded as safe for consumption, making them an attractive choice for a health-promoting remedy. Flavonoids are also readily found in beverages and foods of plant origin, such as fruits, vegetables, cocoa, tea, and wine; hence, they are termed dietary flavonoids [4], contributing meaningfully to disease prevention and treatment [5]. Moreover, flavonoids are known for their ability to modulate various biological processes [5], which can help reduce the risk of disease.

Chrysin (5,7-dihydroxyflavone), a flavonoid found in honey [6], propolis [7], and various medicinal plants and fruits [8,9], plays a role in disease prevention and treatment through multiple mechanisms of action [10]. The pharmacological activities, as well as the therapeutic potential of chrysin were described in a previous study [10].

Recent reports indicate that quercetin and chrysin reduce the levels of pro-inflammatory molecules, including interleukin-6 (IL-6), interleukin-1 (IL-1), and interleukin-10 (IL-10), and tumor necrosis factor (TNF), through the nuclear factor-kappa B (NF-κB) pathway [11]. Another study reported that oral administration of chrysin at 50 mg/kg had a positive effect on bleomycin-induced pulmonary fibrosis. Specifically, chrysin was found to lower hydroxyproline levels, reduce transforming growth factor-beta 1 (TGF-β1) protein expression, reduce lactate dehydrogenase (LDH) activity, and minimize lipid peroxidation [12].

Another study evaluated the chemopreventive efficacy of chrysin against precancerous lesions initiated by N-nitrosodiethylamine (DEN) and induced by Fe-NTA (Ferric Nitrilotriacetate), while also examining its role in regulating inflammation and apoptosis. The results showed that chrysin supplementation inhibited the formation of precancerous lesions by downregulating inflammatory mediators as well as reducing tumor incidence [13]. Another study reported that chrysin has substantial antidiabetic and acute antihyperglycemic effects in diabetic mice. Moreover, IL-1β and TNF-α were diminished, and triglyceride blood levels were reduced [14]

Chrysin is a natural flavonoid belonging to the flavone subclass; it is known for a variety of biological activities, including antioxidant, anti-inflammatory, and anticancer effects. Among the wider flavonoid family, which also includes flavonols and flavanones, chrysin is particularly notable for its unique chemical structure and specific pharmacological effects. These effects encompass significant antioxidant, neuroprotective, and anti-inflammatory properties, all of which have attracted increasing scientific interest. Given the rapid expansion of research concerning chrysin in recent years, a focused review is warranted. While its therapeutic potential appears promising, challenges such as low bioavailability and limited clinical data highlight the need for a more in-depth discussion. These considerations make a separate examination of chrysin justified, rather than treating it within the broader context of flavonoids.

## 2. Methodology

Search engines such as Google, Google Scholar, PubMed, and Scopus were utilized to collect comprehensive information on chrysin and its role in disease management. The primary keywords used included chrysin sources, antioxidant properties, anti-inflammatory effects, cardioprotective activity, and hepatoprotective effects. Additional keywords focused on chrysin’s anti-diabetic, neuroprotective, anti-cancer, anti-microbial, and bone-protective activities. Keywords using chrysin-based nanoformulations in relation to various pathogenesis were also searched. The literature search covered studies published from 1998 to 2025. The literature review comprised research articles and review articles including narrative reviews and systematic reviews, as well as relevant clinical trials on chrysin were included. Moreover, only articles published in the English language were included. While case reports, editorials, theses, and non-English language articles were excluded. 

## 3. Structure, Sources, Pharmacokinetics, Bioavailability and Absorption

**Figure 1:**
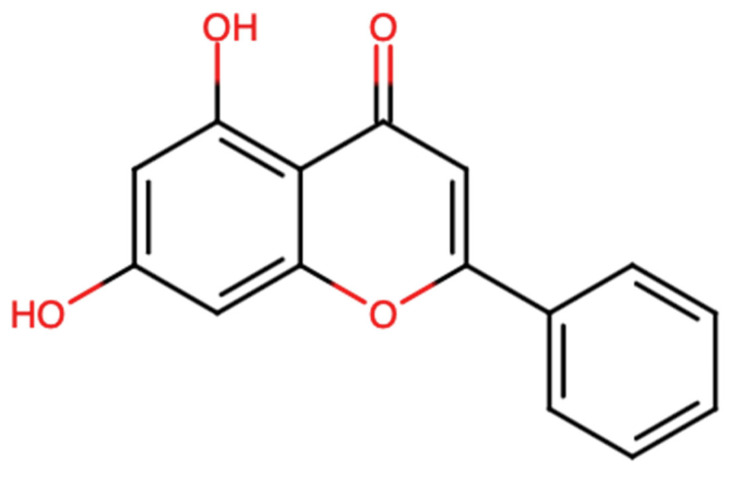
Chemical structure of chrysin (chemical structure modelled using Chemical Sketch Tool: https://www.rcsb.org/chemical-sketch).

Chrysin is a naturally occurring flavonoid in which the two hydroxy groups are located at positions 5 and 7 [Figure 1]. Chrysin has a molecular weight of 254.24 g/mol and the chemical formula C_15_H_10_O_4_. This flavonoid is found in fruits, as well as in vegetable sources. The most eminent natural sources of chrysin comprise honey, with their concentration ranging from 0.10 mg/kg in honeydew honey to 5.3 mg/kg in forest honey [15,16]. Other sources include propolis, which contains 28 g/L of chrysin, and products from stingless bees [17]. Moreover, chrysin is found in Diaphragma juglandis fructus, walnut husks, the blossoms of Juglans regia [18], as well as in the leaves and fruits of doum palms (Hyphaene thebaica) [19]. It is found in edible mushrooms such as *Pleurotus ostreatus* (oyster mushroom) [20].

Pharmacokinetic studies show a decisive role in enhancing the understanding of the pharmacological and toxicological effects of therapeutic compounds. The oral disposition of chrysin was determined in healthy volunteers. A total number of seven subjects received oral doses of 400 mg of chrysin. The levels of chrysin as well as its metabolites were assayed in feces and plasma, and urine. The peak plasma concentrations of chrysin ranged from 3-16 ng mL(-1), with AUC of 5-193 ng mL(-1) h. In contrast, plasma concentrations of chrysin sulfate were noticed to be 30-fold higher. When investigating the urine samples, chrysin and chrysin glucuronide were detected, at 0.2 to 3.1 mg and 2 to 26 mg, respectively. Notably, most of the administered dose appeared in faeces as chrysin. Additionally, parallel experiments conducted in rats demonstrated elevated bile concentrations of chrysin conjugates [21]. The study aimed to make a UPLC-MS/MS method capable of simultaneously quantifying chrysin and its phase II metabolites, and to evaluate their pharmacokinetics in FVB wild-type as well as Bcrp knockout (Bcrp1-/-) mice. The outcomes showed that the sensitive as well as reproducible UPLC-MS/MS method was efficaciously applied to the pharmacokinetic assessment of chrysin in both mouse models following oral administration (20 mg/kg). Although no noteworthy differences were noticed in the systemic exposure of chrysin or its metabolites, the Tmax of chrysin glucuronide was remarkably shorter in Bcrp1-deficient mice. Furthermore, inhibition of BCRP with Ko143 significantly reduced the efflux of chrysin sulfate in Caco-2 cells [22].

The impact of chrysin on various pathological conditions has been confirmed through in vitro studies. However, when applying the in vitro findings to in vivo conditions, several factors come into play, including bioavailability, metabolism, and variations in doses, all of which can affect the results. Chrysin reveals poor absorption, low solubility, speedy metabolism, and fast elimination in human subjects, resultant in very low bioavailability [21,23]. The oral bioavailability of chrysin has been reported to range from 0.003% to 0.02%, with a maximum plasma concentration between 12 and 64 nM [21,24]. Chrysin has an aqueous solubility of 0.06 ± 0.1 mg/mL at pH 6.5 and 0.058 ± 0.04 mg/mL at pH 7.4 [25].

Clinical research has also shown that chrysin’s oral bioavailability is less than 1%, with most of the compound excreted unchanged as aglycone in feces and as chrysin or chrysin-glucuronide in urine [21]. Moreover, because of enterohepatic recycling, chrysin conjugates, specifically chrysin-glucuronide and chrysin-sulfate, are secreted into bile from hepatocytes and transported to the intestine. There, intestinal microbial beta-glucuronidases can quickly hydrolyze these conjugates to release chrysin [26]. A study aims to determine the experimental partition coefficient (Log *P*), and parallel artificial membrane permeability assay (PAMPA), to describe the bidirectional transport of six phenolic compounds, including chrysin in Caco-2 cells. The chrysin’s apical to basolateral permeability is around 5 × 10^−6^ cm/s demonstrating that the passive diffusion rate of chrysin is comparatively fast [25]. Additionally, another study indicated that the intestinal absorption of chrysin is moderate, as Caco-2 permeability within the range of 10 × 10^−6^ cm/s typically suggests that over 75% of chrysin is absorbed in humans [27].

## 4. Effects of Chrysin on Human Health/Disease Management

The role of natural compound in disease prevention and treatment has been established through multiple mechanisms, primarily antioxidant and anti-inflammatory [28]. Research has affirmed its beneficial effects across a range of chronic diseases, supported by both in vivo and in vitro studies that highlight its therapeutic potential. In this article, we will explore the multifaceted role of chrysin in different pathologies, providing a comprehensive overview of how it influences disease processes. By reviewing the current literature, this article aims to provide a comprehensive understanding of chrysin’s role in disease management and its potential as a natural therapeutic agent across various treatment strategies. Here, the chrysin’s roles in different pathogenesis events are described in detail, with the help of previous investigations, as follows:

### 4.1. Anti-Inflammatory Activity

**Figure 2:**
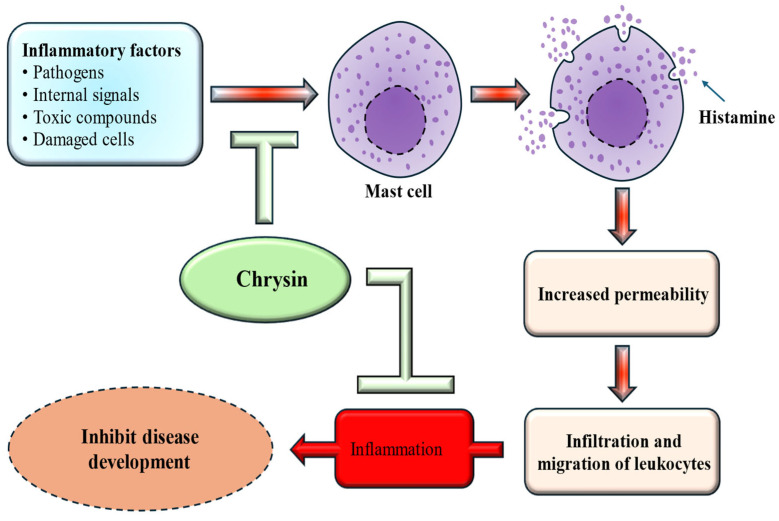
Anti-inflammatory mechanism of chrysin. Chrysin inhibits mast cell activation, thereby reducing histamine release, decreasing vascular permeability, and limiting leukocyte infiltration. This ultimately suppresses inflammation and inhibits disease development.

Inflammation plays a significant role in the onset and progression of many chronic conditions, and the excessive release of inflammatory mediators can suggestively impact overall health. It is closely linked to a wide range of chronic diseases, including neurodegenerative disorders, diabetes, cardiovascular ailments, arthritis, gastrointestinal dysfunction, and cancer. Excessive cytokine production can significantly worsen these health issues. Research has highlighted the potential anti-inflammatory properties of chrysin, which operate through various mechanisms [Figure 2]. The impact of chrysin on human osteoarthritis (OA) chondrocytes was investigated, and the findings showed that chrysin meaningfully inhibited IL-1β–induced IκB-α degradation and suppressed NF-κB activation [29]. Another investigated the impact of chrysin on airway inflammation caused by cigarette smoke. The findings revealed that pretreatment with chrysin effectively inhibited airway inflammation, reduced the release of inflammatory cytokines, and decreased MPO expression [30].

The study findings indicated that chrysin inhibited Inducible Nitric Oxide Synthase (iNOS) expression induced by lipopolysaccharide in a dose-dependent manner at concentrations of 10, 30, and 60 µg/mL. Additionally, chrysin treatment suppressed lipopolysaccharides (LPS)-induced phosphorylation of JAK-STATs, the release of TNF-α and IL-6, as well as the production of reactive oxygen species (ROS) in RAW264.7 cells [31].

The study sought to assess the anti-arthritic properties of chrysin in a preclinical rat model. Treatment with chrysin led to a reduction in arthritis scores, inflammatory cell counts, rheumatoid factor levels, and erythrocyte sedimentation rate, and decreased the mRNA levels of tumor necrosis factor and NF-κB. Furthermore, chrysin diminished the severity of arthritis in the joints, reduced subcutaneous inflammation, lowered inflammatory cell infiltration, and decreased cartilage erosion, bone erosion and pannus formation [32] and it was reported that chrysin attenuated inflammation [33]. Another study revealed that chrysin exhibited anti-inflammatory effects by inhibiting chemokines, nitric oxide, cytokines, and growth factors in macrophages induced by dsRNA [34].

### 4.2. Antioxidant Potential

Oxidative stress occurs when the body cannot adequately neutralize the reactive byproducts formed during cellular metabolism, principally reactive oxygen species (ROS) [35]. Under normal conditions, cells produce low levels of ROS to regulate signaling pathways and help maintain homeostasis [36]. Both ROS and reactive nitrogen species (RNS) exhibit a dual role, with their impact varying based on their concentration levels. When present at high concentrations, they can cause cell and tissue damage, potentially triggering inflammatory diseases [37,38,39,40]. Oxidative stress can lead to inflammation, which subsequently triggers further oxidative stress, generating a vicious circle [41,42]. This ongoing process results in cell damage and fosters a pro-inflammatory environment [43]. Cellular antioxidants like glutathione and antioxidant enzymes, including superoxide dismutase, catalase, and glutathione peroxidase, play a crucial role in sequestering ROS and RNS. This process helps maintain a balanced cellular redox status [44,45].

**Figure 3:**
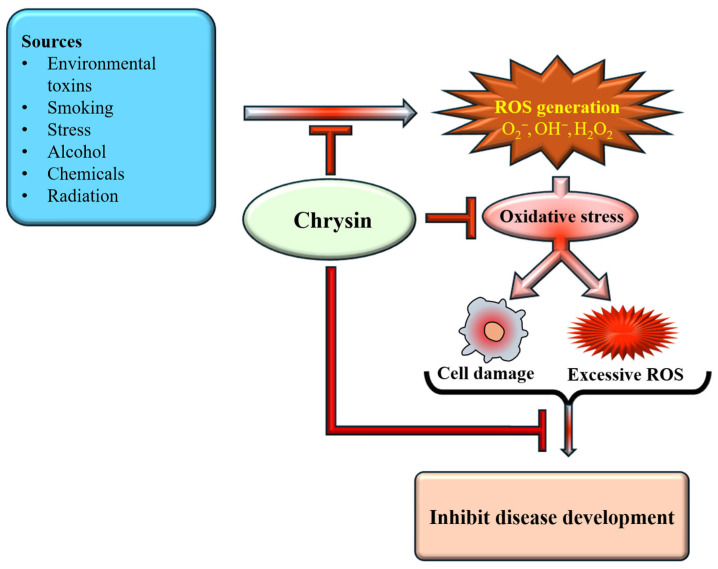
Antioxidant mechanism of chrysin. Chrysin inhibits ROS generation, thereby reducing cell damage. This ultimately suppresses oxidative stress and inhibits disease development.

Natural antioxidants, along with their health benefits, are found in various fruits, seeds, and foods [46]. Flavonoid-rich foods have been extensively studied and are recognized as potent bioactive compounds with diverse biological activities that influence key signaling pathways associated with chronic diseases [47]. The strong antioxidant capacity of natural phenols is mainly attributed to the hydroxyl group, which can effectively quench free radicals, chelate metal ions, induce antioxidant enzyme activity, and regulate the in vivo antioxidant signaling pathways [48]. The study reported that chrysin administration protected the kidneys and liver of rats from oxidative damage induced by chronic ethanol consumption [49]. Research has emphasized the potential antioxidant properties of chrysin, which operate through various mechanisms. Chrysin plays a role in disease prevention through its antioxidant potential [Figure 3].

The protective effects of chrysin against CDDP-induced colon toxicity were explored, showing that chrysin reduced lipid peroxidation, prevented glutathione depletion, and helped maintain antioxidant levels [50]. A study was conducted to assess the impact of chrysin on antioxidant enzyme activity, serum nitric oxide levels, inflammatory cytokines, as well as lipid peroxidation in aging rats. The results indicated that chrysin treatment suggestively reduced elevated levels of lipid peroxidation, inflammatory cytokines, and serum nitric oxide [51]. A study finding concluded that chrysin provides protection against oxidative stress caused by free radicals in rats with hypertension induced by L-NAME [52]. The efficacy of chrysin as a protective agent against propetamphos exposure was assessed. The results showed that in the group receiving both chrysin and propetamphos, noteworthy improvements were noted in oxidative stress indicators, lipid peroxidation levels, antioxidant status, serum biochemical parameters, and histopathological observations [53]. Further research demonstrated that oral administration of chrysin in rats protected against age-related memory decline, increased levels of catalase, superoxide dismutase, and glutathione peroxidase, reduced elevated reactive oxygen species, and helped prevent the decline of brain-derived neurotrophic factor levels in aged mice [54]. Rats with spinal cord injury (SCI) exhibited significant decreases in BBB scores, along with increased spinal cord water content, elevated NF-κB p65, TNF-α, IL-6, IL-1β, iNOS, NO production, and caspase-3 levels. Supplementation with chrysin, however, evidently improved neuronal function recovery and reduced inflammatory markers, as well as inhibiting the iNOS pathway in SCI rats [55].

### 4.3. Hepatoprotective Potential

The liver is an essential internal organ and plays a significant role in our body. It is vital for various physiological functions, such as metabolizing nutrients, detoxifying harmful substances, maintaining balance in lipids, carbohydrates, and proteins, and regulating immune responses [56,57]. Currently, around 4% of global fatalities are caused by liver disease, leading to roughly two million cases [58].

**Figure 4:**
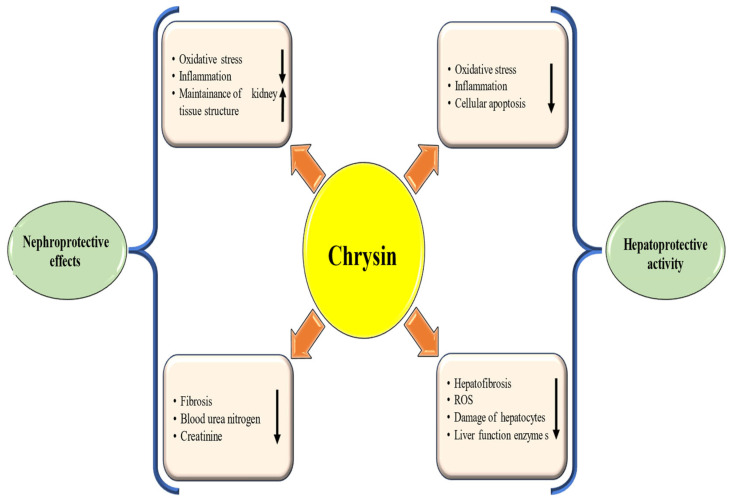
Hepatoprotective and nephroprotective mechanism of chrysin. Chrysin inhibits oxidative stress and inflammation and maintains the tissue architecture. This ultimately suppresses inflammation and oxidative stress and inhibits disease associated with kidney and liver. The downward-pointing arrow shows downregulation, while the upward arrow denotes upregulation.

Natural products have demonstrated hepatoprotective properties, particularly in the prevention and management of liver diseases [59,60]. One of the key ways natural products provide hepatoprotection is by reducing oxidative stress and inflammation. Natural products have antioxidant properties that neutralize free radicals and provide antioxidant defenses. These compounds can also promote liver health by preserving liver tissue architecture and enhancing detoxification processes. They exert their protective effects through various mechanisms, including antioxidant activity, anti-inflammatory properties, reduction of liver enzyme levels, preservation of hepatocytes, and anti-fibrotic potential. Chrysin showed hepatoprotective properties, particularly in the prevention and management of liver diseases through different mechanisms [Figure 4 and Table 1].

An experiment was conducted to measure the hepatoprotective effects of chrysin. The results showed that administering chrysin before carbon tetrachloride (CCl_4_) treatment significantly lowered liver function enzymes. Furthermore, histopathological as well as electron microscopy examinations of liver tissues indicated that pre-treatment with chrysin mitigated the adverse effects of CCl_4_ exposure [61]. A study investigated the protective effects of chrysin on rat liver damage induced by the drugs rifampicin as well as isoniazid. The outcomes showed that administering chrysin at doses of 50, 75, and 100 mg/kg efficiently restored serum biochemical, hematological, lipid, and protein parameters. Additionally, chrysin treatment led to an increase in antioxidant enzyme levels and a decrease in inflammatory cytokines. The study concludes that chrysin protects against rifampicin- and isoniazid-induced oxidative liver injury in rats [62]. Another study reported that chrysin reduced nephro and hepatic damage caused by doxorubicin [63]. Additionally, another study found that administering chrysin to rats with ethanol-induced liver injury resulted in a decrease in thiobarbituric acid reactive substances, lipid hydroperoxides, and conjugated dienes. It also increased the activity of antioxidant enzymes and the levels of vitamins C and E in both tissues and the circulation, compared with ethanol-treated rats that did not receive supplementation [64]. The protective effects of chrysin against hepatotoxicity have been studied. It was found that chrysin lowered liver enzyme levels that had increased due to acetaminophen. Chrysin showed role in inhibiting necrosis and liver injury by reducing TNF-α and liver enzyme levels while enhancing total antioxidant capacity, in that way protecting liver tissue [65]. Pretreatment with chrysin has been shown to prevent hepatotoxicity by improving histopathological changes, reducing oxidative stress, and inhibiting apoptosis [66]. Chrysin was found to mitigate the effects of cisplatin by reducing xanthine oxidase activity, lipid peroxidation, and glutathione depletion. It also lowered the expression of iNOS and cyclooxygenase-2 (COX-2), as well as the levels of TNF-α and NFκB. Additionally, histological results indicated that chrysin protected liver tissue from damage [67]. The study examined how chrysin and quercetin influence the rat liver following TCDD-induced oxidative and histological damage. The findings showed that TCDD significantly increased lipid peroxidation and disrupted the oxidant–antioxidant balance, with corresponding histological changes in the liver. However, treatment with chrysin and quercetin effectively reversed these TCDD-induced oxidative and structural alterations [68]. The study assessed the hepatoprotective effects of chrysin in rats treated with d-galactosamine. Findings showed that chrysin administered at 25, 50, and 100 mg/kg body weight lowered hepatic marker enzyme activities and lipid peroxidation products, while boosting antioxidant enzyme activity and increasing vitamin C as well as E levels [69]. A study reported that intraperitoneal injection of CCl_4_ in rats caused substantial increases in serum liver enzymes and malondialdehyde, along with decreases in reduced glutathione, vitamins C and E, and the activities of superoxide dismutase, catalase, and glutathione peroxidase. In contrast, treatment with chrysin (200 mg/kg body weight) reversed these changes, highlighting its protective effect against CCl_4_-induced oxidative damage in rat tissues [70].

**Table 1:** Mechanisms supporting the hepatoprotective effects of chrysin.

Activity	Study Model	Dose	Outcomes	Refs.
Hepatoprotective potential	CCl_4_-induced liver damage mice model	50 mg/kg	° Treatment with chrysin reduced the activity of enzymes ° Chrysin pre-treatment reduced histopathological changes.	[61]
	Isoniazid- and rifampicin-induced hepatic injury rat model	50, 75, and 100 mg/kg	° The administration of chrysin restored serum biochemical, hematological, proteins, and lipid parameters. ° Administration of CHY, the levels of antioxidant enzymes were also restored. ° The inflammatory cytokines decreased.	[62]
	Doxorubicin-induced hepatotoxicity rat model	40 and 80 mg/kg	° Treatment by chrysin decreased ALT, AST and LDH activity and restores the GSH level ° Pretreatment with chrysin reverted pathological changes	[63]
	Ethanol-induced hepatotoxicity rat model	20 g/kg	° Chrysin administration elevated the activity of antioxidant enzymes and reduced histological changes	[64]
	Acetaminophen-induced hepatotoxicity rat model	40 mg/kg	° Chrysin prevents necrosis and injury by increasing total antioxidant capacity, decreasing liver enzymes and TNF-α ° This compound protects liver tissue.	[65]
	Methotrexate-induced hepatotoxicity rat model	40 and 80 mg/kg	° Pretreatment of chrysin prevents hepatotoxicity by ameliorating histopathological alterations, and oxidative stress	[66]
	Cisplatin-induced hepatotoxicity rat model	25 and 50 mg/kg	° Chrysin ameliorated lipid peroxidation, glutathione depletion, decreased in antioxidant ° Chrysin attenuated expression of iNOS and COX-2, levels of TNF-α and NFκB, and hepatic tissue impairment	[67]
	Tetrachlorodibenzo-p-dioxin-induced hepatotoxicity rat model	50 mg/kg	° The histological and oxidative changes were removed by quercetin and chrysin treatment	[68]
	D-galactosamine-induced hepatitis rat model	25, 50 and 100 mg/kg	° Chrysin treatment led to a reduction in hepatic marker enzyme activities ° It also resulted in an increase in the activities of free-radical scavenging enzymes.	[69]
	CCl_4_-induced tissue injury rat model	200 mg/kg	° The chrysin improved the activities of antioxidant enzymes	[70]

### 4.4. Nephroprotective Effects

Renal injury can arise from various factors, including oxidative stress, inflammation, exposure to toxins/chemicals, and hypertension. These factors can disrupt kidney structure and function, leading to cellular damage and the progression of the disease.

Natural products have demonstrated important nephroprotective properties, making them valued in both the prevention and management of renal diseases [71,72]. Moreover, Flavanoids, including hesperedin, myricetin, and quercetin, play a crucial role in preventing renal injury through their antioxidant and anti-inflammatory effects, as well as their ability to protect cells from injury [73,74]. Natural products, including 6-Gingerol and curcumin, have demonstrated nephroprotective properties, particularly in the prevention and management of renal diseases [75,76]. Chrysin was studied for its effects on adenine-induced chronic kidney disease in rats, where adenine decreased creatinine clearance and increased levels of creatinine, urea, inflammatory cytokines, and renal damage. Chrysin, especially at higher doses, moderately mitigated these adverse changes, including inflammation and fibrosis [77]. Chrysin showed nephroprotective properties, particularly in the prevention and management of renal diseases through different mechanisms [Figure 4 and Table 2]. The role of chrysin against the harmful effects of pemetrexed on kidney tissue was examined. In the pemetrexed + chrysin group, BUN (Blood Urea Nitrogen), MDA, creatinine and total oxidant status were decreased, Superoxide dismutase (SOD) and TAS were increased compared to the pemetrexed group [78]. The effect of chrysin against nephrotoxicity in rats was checked. The renal protective effect of chrysin was related to increasing the antioxidant enzyme activities and decreasing the regulation of serum renal toxicity markers. Chrysin therapy meaningfully reduced inflammatory markers [79]. Chrysin has been shown to mitigate lead acetate (PbAc)-induced kidney toxicity by decreasing lipid peroxidation and boosting antioxidant enzyme activity. It also protects DNA from PbAc’s harmful effects and improves mineral levels in renal tissue, indicating significant protective effects against kidney damage [80]. Chrysin has been shown to mitigate 5-Fluorouracil (5-FU)-induced renal toxicity in rats by reducing serum toxicity markers and enhancing antioxidant defenses while regulating apoptosis. Histopathological analyses support these biochemical findings, suggesting that chrysin could be an effective modulator in mitigating 5-FU-caused renal toxicity [81]. Another study result demonstrated that chrysin and hesperidin suggestively increased antioxidant enzyme levels, decreased the inflammatory parameters and levels of MDA against renal injury. Chrysin, as well as hesperidin, alleviated renal injury induced by colistin via anti-inflammatory and antioxidant activities [82].

Another study reported that cisplatin induced acute kidney injury, evidenced by increases in blood urea nitrogen, MDA, and serum creatinine, along with a marked reduction in creatinine clearance. Chrysin administration reversed these cisplatin-induced alterations in a dose-dependent way, and histological analyses further confirmed these protective effects [83]. Pretreatment with chrysin effectively reduced renal oxidative damage by decreasing DNA damage and lowering blood urea nitrogen, creatinine, lipid peroxidation, and xanthine oxidase activity. Additionally, it enhanced both enzymatic and non-enzymatic antioxidant levels, with histological findings further confirming chrysin’s protective effects against cisplatin-induced renal injury [84]. The study investigated the protective effects of chrysin against gentamicin-induced nephrotoxicity, which led to decreased urine flow and increased serum creatinine and urea levels. Chrysin treatment improved renal tissue outcomes by reducing inflammation and oxidative stress markers, restoring antioxidant activity, and normalizing renal function, as evidenced by improvement in histopathological and biochemical parameters [85].

**Table 2:** Chrysin shows nephroprotective activity of through different mechanisms.

Activity	Study Type	Dose	Outcome of the Study	Refs.
Nephroprotective effects	Adenine-induced chronic kidney diseases rat model	10, 50 and 250 mg/kg	° Adenine-induced activities such as lowered creatinine clearance and raised the concentrations of urea, creatinine were mitigated by chrysin ° Increased inflammatory cytokines and renal fibrosis decreased by chrysin treatment	[77]
	PMTX-induced nephrotoxicity rat model	50 mg/kg	° Chrysin ameliorative nephrotoxicity Chrysin reduced oxidative damage	[78]
	Paracetamol-induced nephrotoxicity rat model	25 or 50 mg/kg	° Chrysin showed antioxidants, anti-inflammatory activities	[79]
	Lead acetate-induced renal toxicity rat model	25 and 50 mg/kg	° Chrysin showed anti-inflammatory and anti-apoptotic effects	[80]
	5-Fluorouracil-induced renal toxicity rat model	50 and 100 mg/kg	° Treatment by chrysin decreased serum toxicity markers, increased antioxidant armory and improved histopathological changes	[81]
	Colistin-induced renal injury rat model	25 and mg/kg	° Chrysin reduces colistin-induced nephrotoxicity through anti-inflammatory and antioxidant activities	[82]
	Cisplatin-induced renal failure rat model	20 and 40 mg/kg	° Cisplatin-induced renal damage and chrysin effectively restoring renal function.	[83]
	Cisplatin-induced nephrotoxicity rat model	25 and 50 mg/kg	° Pretreatment with chrysin attenuated renal oxidative damage and toxicity markers	[84]
	Gentamicin-Induced Renal Injury Rat model	100 mg/kg	° The chrysin treatment w protected renal tissues and caused improvement in histopathological damage	[85]

### 4.5. The Potential Role of Chrysin in Different Cancers

**Figure 5:**
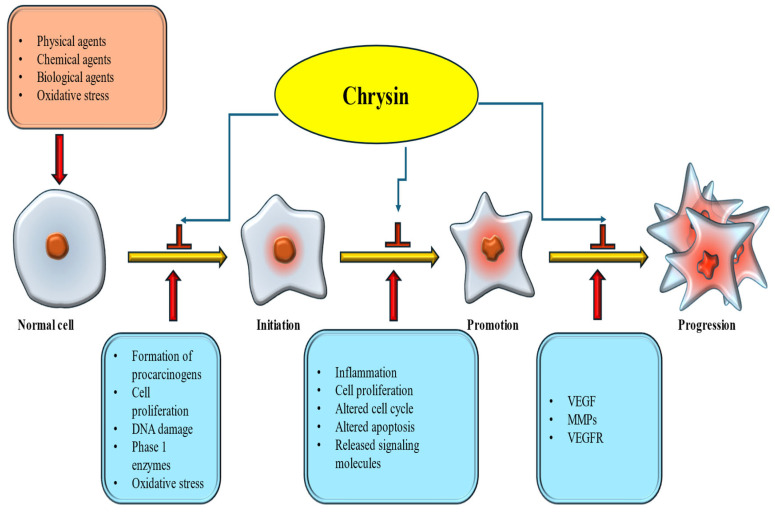
Ani-cancer mechanism of chrysin. Chrysin inhibits initiation and promotion steps, and finally preventing cancer formation.

Cancer develops through a multi-step process. It begins with Initiation, where normal cells undergo genetic alterations due to exposure to carcinogens. This is followed by promotion, in which the initiated cells start to divide abnormally. The final stage is progression, where these abnormal cells accumulate additional mutations, grow uncontrollably, and invade surrounding tissues. Natural products and their bioactive constituents affect cancer development and progression by regulating multiple cell-signaling pathways [86,87]. Chrysin is a prominent flavonoid whose potential anticancer effects have been recognized through multiple mechanisms, primarily by inhibiting cancer initiation and progression, thereby slowing or preventing tumor development [Figure 5].

Furthermore, chrysin has been reported to inhibit cancer cell proliferation by modulating various cellular and signaling pathways. Its effects on different types of cancer involve multiple mechanisms, including the induction of apoptosis, cell cycle arrest, and the regulation of key signaling pathways, as delineated below.

#### 4.5.1. Colorectal Cancer

Colorectal cancer is a substantial global health concern, being one of the leading causes of cancer. Conventional cancer treatment methods, for instance, surgery, hormone therapy, chemotherapy, and targeted therapies, face challenges with drug resistance, systemic toxicity, and insufficient selectivity for cancer cells [88,89,90]. Chrysin played a decisive role in preventing colorectal cancer by modulating oxidative stress, inflammation, cell proliferation, apoptosis, and various cell signaling pathways [Table 3].

Chrysin demonstrated a similar level of cell viability inhibition in human colorectal cancer (CRC) cells, comparable to that of the 5-FU combination treatment. These findings imply that chrysin could serve as a potential alternative to the oxaliplatin and 5-FU combination for achieving antitumor effects through autophagy in CRC therapy [91].

Chrysin exhibited a dose-dependent cytotoxic effect on CT26 colon cancer cells. Biological assays indicated that chrysin induced cytotoxicity in cancer cells primarily by triggering apoptosis. Flow cytometry analysis indicated that the percentage of annexin-positive cells increased progressively with higher concentrations of chrysin. Specifically, the percentage of apoptotic cells was found to be 0.7% in the control group, while it rose to 77.2% at a concentration of 80 µg/mL and reached 85.7% at 100 µg/mL in the treated groups. Colorimetric assays for caspase-3 and caspase-9, along with an examination of Bax expression, demonstrated the involvement of the intrinsic apoptotic pathway in chrysin’s cytotoxic effects. Additionally, in vivo assays showed that oral administration of chrysin at 8 mg/kg and 10 mg/kg resulted in a significant reduction in tumor volume compared with the control group receiving the vehicle solution [92].

Another study reported that chrysin, as well as daidzein treatment, restored the biochemical changes and returned histopathological results close to the normal status. Also, chrysin and daidzein showed anticancer activity against SW620 cells, associated with reduced protein expression of p-AKT/AKT and p-ERK/ERK. This study concludes that chrysin and daidzein may have potential anticancer properties in the treatment of colon cancer [93].

#### 4.5.2. Gastric Cancer

Natural products show an important role in preventing gastric cancer by modulating numerous critical biological processes. They help reduce inflammation, regulate cell proliferation, promote apoptosis, and affect various cell signaling pathways. A study demonstrated that chrysin meaningfully induced the expression of TET1 in GC cells. Furthermore, outcomes advised that chrysin markedly inhibit cell migration and invasion. The results of the cell apoptosis showed that apoptosis was meaningfully elevated in MKN45 cells treated with chrysin compared to the control group. This study demonstrated that chrysin exhibited anti-tumor effects by regulating TET1 expression in gastric cancer (GC) and identified TET1 as a novel and promising therapeutic target for GC treatment [94]. Chrysin suggestively inhibited endogenous as well as inducible RON expression in a dose-dependent manner. The results indicate that chrysin exerts anticancer effects, at least by inhibiting RON expression through the suppression of Egr-1 and NF-κB in AGS cells associated with gastric cancer [95].

The study aimed to examine the expression levels of miR-9, miR-18a, miR-21, miR-221, miR-22, miR-34a, and miR-126 to assess the anti-cancer effects of chrysin. The results indicated that miR-9, miR-22, miR-34a, in addition miR-126, were upregulated, whereas miR-221, miR-18a, and miR-21 were downregulated in the gastric carcinoma cell line [96]. The study findings designate that chrysin might contribute to its anticancer effects by regulating MMP-9 expression. This regulation occurs through the suppression of AP-1 activity, which is achieved by inhibiting the JNK1/2 and ERK1/2 signaling pathways in AGS gastric cancer cells [97].

#### 4.5.3. Esophageal Cancer

Esophageal cancer is among the most predominant malignancies in the gastrointestinal system worldwide. In 2020, around 604,000 new cases and 544,000 deaths were documented, as noted in global cancer statistics [98]. Esophageal cancer represents the seventh-highest incidence of cancer and stands as the sixth-leading cause of cancer-related deaths worldwide [98]. Chrysin play a crucial role in preventing esophageal cancer by influencing numerous key processes [Table 3]. The cytotoxic effects of structurally associated flavones and flavonols were evaluated on a human esophageal squamous cell carcinoma cell line. The MTT assay results demonstrated that flavones (apigenin, luteolin, chrysin) and flavonols (quercetin, myricetin, kaempferol) induce cytotoxicity in a manner dependent on concentration and exposure time. The relative cytotoxic potency of these compounds was observed in the following sequence: luteolin > quercetin > chrysin > kaempferol > apigenin > myricetin [99]. Chrysin treatment led to a dose-dependent reduction in the viability of esophageal squamous cell carcinoma (ESCC) cell lines. However, it exhibited minimal cytotoxic effects on normal esophageal epithelial cells (SHEE) at the same concentration levels used in ESCC cell lines. Additionally, chrysin significantly disrupted the DGKα/FAK signalosome, inhibiting FAK-controlled signaling pathways and hindering the malignant progression of ESCC cells in both in vitro and in vivo studies, without causing any toxicity to normal cells. Chrysin reduced the growth of ESCC tumors in a dose-dependent way and increased the expression of cleaved PARP and caspase 3. Histological analysis of heart, kidney, liver, spleen, as well as tissues revealed no differences between the control group and the chrysin treatment groups, indicating that chrysin did not cause any toxic effects in normal tissues [100].

#### 4.5.4. Pancreatic Cancer

Pancreatic cancer (PC) is difficult to detect and treat, making it one of the deadliest types of cancer. Currently ranked as the seventh leading cause of cancer-related deaths worldwide, it is projected to rise to the most common cause of cancer-related death in 2040 [101]. Chrysin plays a crucial role in preventing this cancer by influencing numerous key processes. It plays a role in the reduction of inflammation, regulating cell proliferation, promoting apoptosis, and affecting various cell signaling [Table 3].

Chrysin induced cell cycle arrest and meaningfully reduced cell viability. When chrysin was combined with 17β-estradiol, its inhibitory effect on cell proliferation was further enhanced. In vivo studies showed that treatments with chrysin (50 mg/kg, p.o.) as well as G1 (10 mg/kg, i.p.) meaningfully hindered tumor growth. To assess physiological changes in tumor tissues, the expression levels of c-Myc and Ki-67 were analyzed. Both c-Myc and Ki-67 expressions were found to be suppressed in the tumor tissues treated with chrysin and G1-treated tumor tissues [102].

Study using the GEPIA database examined tumor tissue samples from PC patients and found that CBR1 was more highly expressed in PC tissues and was meaningfully associated with clinicopathological characteristics of PC. Additionally, studies have shown that chrysin directly interacts with CBR1, inhibiting its enzymatic activity at both the molecular and cellular levels. This inhibition led to elevated intracellular ROS levels, triggering ROS-dependent autophagy. Overall, the findings indicate that chrysin enhances pancreatic cancer cell sensitivity to gemcitabine by inducing ferroptosis death, both in vitro and in vivo [103].

#### 4.5.5. Liver Cancer

Natural compounds have shown important potential in inhibiting liver pathogenesis, suggesting an auspicious avenue for therapeutic development. These compounds possess anti-inflammatory, antioxidant, and antiviral properties that protect the liver and prevent disease progression. This flavonoid showed a role in liver cancer prevention and treatment [Table 3]. A study on hepatocellular carcinoma demonstrated that chrysin inhibits the proliferation of human hepatocarcinoma cells in a dose-dependent manner. Furthermore, chrysin caused induction of cell cycle arrest in the G2 phase [104]. Chrysin efficiently inhibits tumor growth and boosts anti-tumor immunity in mice, demonstrated by a higher proportion of CD4^+^ and CD8^+^ T cells within tumor tissues in the H22 xenograft model. In addition, chrysin markedly downregulates PD-L1 expression both in vitro and in vivo [105]. A study found that chrysin, or its derivative, has promising potential as a novel treatment for hepatocellular carcinoma (HCC) by suppressing glycolysis and inducing apoptosis [106]. To assess the impact of chrysin on the self-renewal ability of HCC cells, MHCC97H and SMMC-7721 cells were exposed to various concentrations of chrysin (0.0, 10.0, 20.0, 40.0 µM) and subsequently cultured using sphere-forming culture. The results indicated that chrysin reduced the sphere-forming rate in a dose-dependent manner in both SMMC-7721 and MHCC97H cells. Additionally, SHP-1 expression levels increased with higher concentrations of the drug in both cell lines. Inclusive, the study results confirmed that chrysin acts as a candidate for the treatment of HCC via modulating SHP-1/STAT3 signaling pathway [107].

#### 4.5.6. Breast Cancer

Breast cancer is one of the most common malignancies worldwide and remains a leading cause of cancer-related mortality among women [108]. Despite technological advances in early detection, for example, mammography, computed tomography, magnetic resonance imaging, ultrasound, and positron emission tomography [109], patients with breast cancer continue to face a substantial lifelong risk of metastasis, attributable to the inherently aggressive nature of breast cancer [110]. Alarmingly, approximately 30% of individuals diagnosed at an early stage eventually experience metastatic relapse [111,112]. Current treatments for breast cancer can be expensive and may result in unwanted side effects. In this context, natural products have emerged as important alternatives for managing this pathogenesis, offering various mechanisms of action that can complement traditional therapies. These natural compounds may help reduce tumor growth, enhance the immune response, and mitigate side effects, presenting a promising approach to breast cancer treatment. This flavonoid showed a role in breast cancer prevention and treatment [Table 3]. The impact of chrysin on MCF-7 cell survival was investigated, revealing a dose-dependent reduction in cell viability, suggesting a toxic effect. It was hypothesized that treatment with chrysin leads to the loss of genomic integrity, demonstrating impairment of genomic stability in MCF-7 cells. Additionally, it was established that chrysin also destabilizes genomes and decreases cell survival in another breast cancer cell line, BT474. Furthermore, chrysin significantly inhibited the recruitment of 53BP1 to sites of DNA damage. Subsequently, the effect of chrysin on the sensitization of MCF-7 cells to etoposide was assessed. The results indicated that the combination of the two treatments synergistically decreased the survival of MCF-7 cells (using 5 µM chrysin and 0.3 µM etoposide), suggesting that chrysin may have therapeutic potential in breast cancer by acting as a sensitizer to etoposide [113]. Another study reported that treating MDA-MB-231 cells with chrysin in combination with radiation therapy (RT) resulted in synergistic antitumor effects. The findings indicated that chrysin enhanced RT-induced apoptosis in MDA-MB-231 cells compared with either treatment alone (chrysin or RT). Moreover, the HIF-1α expression was diminished in the cells subjected to the combined therapy. The apoptotic effect of combinational therapy was correlated with reduced expression of Bcl-2, increased Bax, and p53 levels [114]. A study examined the immunomodulatory effects of chrysin on Jurkat-T and NK-92 cells in the context of targeting breast cancer cells. Chrysin promoted activation of NK-92 and T cells, mainly when supplemented with human recombinant PHA-M and IL-2. Furthermore, the findings indicated that chrysin meaningfully enhanced the cytotoxicity of both T cells as well as NK-92 cells against MDA-MB-231 and MCF-7 breast cancer cell lines, with the more significant effect noticed in MCF-7 cells [115]. The activity of chrysin as well as quercetin towards MCF-7 as well as MDA-MB-231 breast cancer cells was examined. Both compounds (chrysin and quercetin) inhibited cellular proliferation in a dose-dependent way. Both compounds association made cell cycle arrest in the sub-G0/G1 phase in MDA-MB-231 cells [116]. Chrysin reduced the survival of 4T1 cells after exposure to hypoxia (1% O_2_). Daily chrysin oral administration in mice implanted with 4T1 cells meaningfully suppressed lung metastatic colonies growth. Furthermore, the antimetastatic action of the DR5 mAb was greater when combined with chrysin [117]. The outcome of the study reported that the combination of chrysin and metformin had high synergistic properties in killing cancer cells [118]. Another study result demonstrated that MTT assay-based findings exhibited that chrysin had an anti-proliferative activity on MCF-7 cells in a dose- and time-dependent way. Moreover, chrysin induced apoptosis in MCF-7 cells. Chrysin inhibits the growth of the breast cancer cells through the induction of cancer cell apoptosis, which may, in part, describe its anticancer action [119]. The study results designated that chrysin significantly reduced both migration as well as invasion of TNBC cells [120].

#### 4.5.7. Cervix Cancer

Cervical cancer is the most frequently diagnosed cancer among women. Even with progress in screening and treatment options, the mortality rate for cervical cancer continues to be substantial, highlighting the critical need for new therapeutic approaches. Current treatments for cervical cancer can be expensive and may result in unwanted side effects. In this condition, natural products have emerged as important alternatives in managing this pathogenesis, offering various mechanisms of action that could complement traditional therapies. This flavonoid showed a role in cervical cancer prevention and treatment as described in Table 3. An experiment was conducted to assess the pro-apoptotic as well as antiproliferative effects of chrysin on human cervical cancer cells. Treatment of HeLa cells with chrysin resulted in a dose- and time-dependent decrease in cell viability, accompanied by clear evidence of DNA fragmentation and altered nuclear morphology. Chrysin treatment increased the expression of proapoptotic genes and caspases, while reducing the transcript levels of anti-apoptotic and cell cycle regulatory genes. Additionally, chrysin upregulated pro-apoptotic proteins and downregulated anti-apoptotic proteins, supporting chrysin-induced apoptosis in HeLa cells. Furthermore, chrysin inhibited cell proliferation and induced apoptosis by modulating AKT/MAPK pathway genes and multiple apoptotic genes [121]. Another study was performed to observe chrysin-induced epigenetic changes in HeLa cells. The findings revealed that chrysin enhances cytostatic activity and suppresses HeLa cell migration in a dose- and time-dependent manner [122].

#### 4.5.8. Endometrial Cancer

Endometrial cancer is the most commonly diagnosed cancer among gynecological cancers [123]. In terms of risk factors, various non-genetic traits and medical conditions have been linked to the development of endometrial cancer [124]. Medicinal plants have been recognized for their potential role in cancer prevention, including endometrial cancer, by various mechanisms. These plants contain numerous phytochemicals that contribute to their chemopreventive properties. In this view, chrysin plays a substantial role in the management of this cancer.

A different study explored the potential of chrysin to induce apoptosis in EC cells. The examination of cell apoptosis revealed that chrysin significantly raised the proportion of apoptotic cells. Western blot analysis demonstrated that treating Ishikawa and HEC-1A cells with chrysin resulted in a reduction of Bcl-2 expression and an increase in Bax levels. These findings indicate that chrysin promotes apoptosis in EC cells. Additionally, transmission electron microscopy (TEM) images revealed the presence of autophagosomes and autophagolysosomes in the chrysin-treated group, which are characteristics of autophagic cells. Notably, in cells pre-treated with 5 µM CQ before the application of 40 µM chrysin, these effects were further enhanced. It was shown that chrysin leads to the accumulation of reactive oxygen species (ROS) in cells, which may contribute to the autophagy induced by chrysin in EC cells. Furthermore, it was observed that the inactivation of the Akt/mTOR signaling pathway by chrysin played a role in the activation of autophagy in these cells [125].

#### 4.5.9. Ovarian Cancer

Ovarian cancer treatments are costly and come with unwanted side effects. In this context, natural products reduce tumor growth, boost the immune response, and alleviate side effects, making them a compelling option for enhancing ovarian cancer treatment.

The study aimed to assess the functional impact of chrysin on ovarian cancer progression using the OV90 and ES2 cell lines. The results confirmed that chrysin suppressed ovarian cancer cell proliferation and promoted cell death by elevating ROS levels, increasing cytoplasmic Ca^2+^ concentrations, as well as inducing mitochondrial membrane potential loss [126].

#### 4.5.10. Prostate Cancer

Prostate cancer is one of the deadliest cancer types for males [127]. Various etiological factors play a significant role in the development and progression of this cancer. Factors, for example, advanced age, African descent, and a family history of the disease, are linked with poorer prognoses for this cancer [128]. In this scene, chrysin plays an imperative role in the management of this cancer.

The impact of chrysin on the progression of prostate cancer cells was examined using the PC-3 and DU145 cell lines. The findings revealed that chrysin promoted apoptosis in these cells, leading to an increased population of both cells in the sub-G1 phase of the cell cycle. Additionally, chrysin decreased the expression of proliferating cell nuclear antigen in the prostate cancer cell lines when compared to untreated cells. Furthermore, chrysin caused an enhanced production of reactive oxygen species (ROS) and lipid peroxidation and loss of mitochondrial membrane potential in a dose-dependent manner. Together, these outcomes designate that chrysin initiates cell death through induction of mitochondrial-mediated apoptosis as well as ER stress, and of signaling pathways regulation accountable for proliferation of prostate cancer cells [129]. The PC-3 prostate carcinoma cell line was treated with chrysin to assess its cytotoxicity. A substantial inhibition of cell proliferation was noticed only at the highest concentration (40 µM). In addition, cells exposed to 40 µM chrysin exhibited pronounced cell shrinkage and greater growth suppression compared with those treated with the lower dose (10 µM) [130].

#### 4.5.11. Bladder Cancer

The current mode of treatment for this type of cancer is not only expensive but also comes with a range of negative effects that can meaningfully impact a patient’s quality of life. An alternative line worth considering is the use of phytochemicals, which have revealed promise in both the treatment as well as prevention of various cancers, including bladder cancer. This flavonoid showed a role in bladder cancer prevention and treatment through various mechanisms [Table 3 and Figure 5].

Recent studies indicate that chrysin plays a significant role in inducing apoptosis. Additionally, chrysin has been shown to decrease the expression levels of anti-apoptotic B cell lymphoma (Bcl) proteins while promoting the expression of pro-apoptotic Bcl-2 associated X. Collectively, these findings suggest that chrysin could be a promising therapeutic candidate for targeting bladder cancer [131]. Another finding reported that the chrysin treatment decreased the cell viability as well as caused apoptosis. Furthermore, in the *TP53*-mutated cell lines, chrysin controlled the expression of the *HDAC*, *DNMT1* as well as, *HAT1* epigenetic genes, which might be a plus to the death detected in the cells with p53 mutation [132].

#### 4.5.12. Renal Cancer

Renal cancer poses substantial challenges in treatment as well as management. However, several natural compounds have shown potential for combating renal cancer through various mechanisms. A study was performed to explore the efficacy of chrysin as an anticancer agent. Renal cancer was developed by N-nitrosodiethylamine and promoted by the administration of ferric nitrilotriacetate (Fe-NTA). The outcomes of the study reported that the chemopreventive effects of chrysin against renal hyperproliferative responses, oxidative stress, and inflammation induced by Fe-NTA, as well as its influence on two-stage renal carcinogenesis. Pretreatment of animals with chrysin noticeably inhibited all. Additionally, administering chrysin prophylactically to animals prior to Fe-NTA exposure was powerful in modulating markers of oxidative stress as well as renal damage, leading to a reduction in the damage caused by Fe-NTA [133].

#### 4.5.13. Bone Cancer

A study based on osteosarcoma reported that chrysin synergistically enhanced the cytotoxic properties of tumor necrosis factor-related apoptosis-inducing ligand (TRAIL). Chrysin sensitize cells against the TRAIL-induced apoptosis, increases the caspase 8 activity and this result is accomplished by reducing the expression levels of anti-apoptotic genes. These findings advocate that chrysin sensitize the osteosarcoma cell lines against TRAIL via the death receptor pathway induction. In addition, a combination therapy involving these agents may represent an effective treatment approach to enhance the clinical effectiveness of TRAIL-induced apoptosis in patients with osteosarcoma [134].

#### 4.5.14. Thyroid Cancer

The antiproliferative effects of chrysin on anaplastic thyroid cancer (ATC) cells were evaluated. Chrysin treatment suppressed the proliferation of HTH7 as welll as KAT18 cells in a dose- and time-dependent way. Moreover, chrysin increased the Bax/Bcl-2 expression ratio in ATC cells following treatment [135]. The anti-tumor activity of chrysin on Anaplastic thyroid carcinoma cells in vitro was investigated, and it was reported that chrysin treatment suppressed growth and induced apoptosis. PUMA and Notch-1 were activated, and Slug was inactivated by chrysin treatment [136].

A study result reported that chrysin treatment of ATC cells resulted in a dose-dependent reduction in cellular growth. Both Notch1 protein and messenger RNA levels, as well as its downstream effector Hes1, were up-regulated following treatment. In the chrysin treatment group, the growth of ATC xenografts was significantly inhibited. Specifically, the average tumor volume increased 11-fold in the vehicle control group after 21 days of DMSO treatment. In contrast, chrysin-treated animals observed an average of only a 4.4-fold increase in tumor growth. Overall, chrysin led to a reduction in ATC xenograft growth by approximately 59% compared to the control group [137].

#### 4.5.15. Lung Cancer

Lung cancer remains an important cause of worldwide mortality, affecting both men and women with a concerningly low survival rate as compared to other cancers [138]. The existing treatment for this type of cancer tends to be costly and often leads to several adverse effects that can significantly affect a patient’s quality of life. An alternative approach worth exploring is the use of phytochemicals, which have shown potential in both treating and preventing various forms of cancer, including lung cancer. This flavonoid showed a role in lung cancer prevention and treatment through various mechanisms [Table 3 and Figure 5]. Chrysin was shown to inhibit cell growth as well as induce apoptosis in cultured lung cancer cells, and its treatment led to a marked activation of AMPK in these cells [139]. It was demonstrated that chrysin enhanced the inhibitory effects of TRAIL in comparison to TNF-α on cell viability in lung cancer cells and changed the nuclear morphology of cells, and treatment with chrysin increases TRAIL-induced apoptosis [140]. Cell viability diminished in a dose- and time-dependent means in malignant cells treated with either honey as well as chrysin, in comparison with the non-malignant cells. Moreover, it was reported that chrysin induced apoptosis in the lung cancer cells [141]. Another study reported that quercetin, chrysin, curcumin, luteolin, and apigenin meaningfully blocked the promoting properties of NiCl_2_ (Ni) on migration as well as invasion in A549 and H1975 human lung cancer cells [11].

#### 4.5.16. Oral Cancer

Natural compounds have gained increasing consideration as potential preventive as well as therapeutic agents in oral cancer. Natural products are described to have chemopreventive as well as anti-tumor activities for oral cancer treatment [142]. Chrysin flavonoid, showed a role in oral cancer prevention and treatment [Table 3]. A study was designed to examine the anticancer potential of purified chrysin in oral squamous cell carcinoma (OSCC) cell lines. Chrysin-treated HSC4 cells showed a gradual decline in cell viability when exposed to chrysin concentrations ranging from 50 to 1000 µM. Similarly, the viability of chrysin-treated SCC25 cells also decreased progressively within the same concentration range. It has been suggested that chrysin concentrations of 100 and 200 µM have the potential to inhibit cell proliferation [143]. Another result finding demonstrated that chrysin meaningfully stimulates the cytotoxic activity of cisplatin. Also, this combined treatment noticeably downregulated the antioxidant enzyme expression and significantly improved ROS levels [144]. The role of chrysin on the apoptosis of the oral squamous carcinoma KB cell line was studied. KB cells were treated with varying concentrations of chrysin (1, 2, 4, 8, 16, as well as 32 µmol/L). The results showed that chrysin inhibited KB cell proliferation in a concentration-dependent manner, accompanied by an increase in apoptosis, a reduction in mitochondrial membrane potential, activation of caspase-3/7, and a decrease in the phosphorylation of AKT and PI3K [145].

#### 4.5.17. Brain Cancer

Medicinal plants have gained increasing consideration as potential alternative approaches in brain cancer prevention through suppression of cancer cell proliferation and regulation of the cell cycle. A study was conducted to assess the antitumor role of chrysin in glioblastoma cells. The findings showed that chrysin inhibited glioblastoma cell proliferation, migration, and invasion in a dose- and time-dependent way [146]. Another study explored the relationship between mitogen-activated protein kinase (MAPK) signaling pathways and chrysin-induced growth inhibition in rat C6 glioma cells. The results showed that chrysin impeded cell-cycle progression at the G1 phase in both a dose- and time-dependent manner. Additionally, chrysin treatment significantly reduced Rb phosphorylation levels in C6 glioma cells [147].

#### 4.5.18. Skin Cancer

Medicinal plants are increasingly recognized as effective alternatives for skin cancer prevention, demonstrating their ability to suppress cancer cell proliferation and regulate cell signaling molecules. Chrysin has been shown to play a role in skin cancer prevention and treatment [Table 3]. The study was designed to explore the anticancer role of chrysin on human melanoma cells A375P and A375SM. The findings revealed that chrysin efficiently inhibited the viability of these cell lines by promoting apoptosis as well as autophagy. Both cell lines treated by chrysin displayed the presence of autophagic vacuoles and acidic vesicular organelles. Furthermore, chrysin was revealed to decrease the phosphorylation of mTOR/S6K pathway proteins, suggesting that this pathway plays a role in mediating chrysin-induced apoptosis as well as autophagy in both cells [148]. The study results indicated that chrysin effectively inhibited ankios resistance migration, invasion, and suppressed the tube formation ability of melanoma cells. The findings concluded that chrysin treatment can reduce the metastatic rate of melanoma by regulating FOXM1/β-catenin signaling. This suggests the potential application of chrysin for melanoma therapy [149].

#### 4.5.19. Leukemia

The study exhibited that chrysin at concentrations of 5–50 µM reduced the cell viability in a concentration- as well as time-dependent way. An in vivo study was conducted using WEHI-3 leukemic BALB/c mice to evaluate the antileukemia effects of chrysin. It was reported that chrysin increased the percentage of CD19, CD3, and Mac-3 cell surface markers in treated mice. Furthermore, there was a noteworthy increase in phagocytosis by macrophages in leukemic mice after treatment with chrysin [150].

**Table 3:** Chrysin’s role in the prevention and treatment of cancer by various mechanisms.

Cancer	Study Model (In Vivo & In Vitro)	Dose	Outcome	Refs.
Colon cancer	CT26 tumor cells in BALB/c mice	0.5–10 mg/kg	° The oral administration of chrysin (8 and 10 mg·kg^−1^) showed regression in the tumors’ volume	[92]
	CT26 cells	40, 80, 100 µg·mL^−1^	° Chrysin exerted its cytotoxicity effects through intrinsic apoptotic pathway ° Chrysin induced chromatin condensation	[92]
	DMH-induced colorectal cancer model	125 and 250 mg/kg	° Chrysin treatment restored the biochemical alterations ° It reverted histopathological changes	[93]
Gastric cancer	MKN-45 cells	40 µM	° By treatment with chrysin TET1 expression ° Chrysin altered cell migration and invasion	[94]
	MKN45 cells injected into nude mice	20 mg/kg	° Chrysin inhibited tumor growth	[94]
	AGS cells	0–60 µM	° Chrysin inhibits PMA-induced RON	[95]
	AGS cells	10–50 µM	° Chrysin inhibits MMP-9 activity	[97]
Esophageal cancer	KYSE150, KYSE30, KYSE410, KYSE450, YSE2 cells	10, 25, and 50 µmol/L	° Chrysin promotes the apoptosis and inhibits the FAK/AKT pathway	[100]
	ESCC cells inoculated into nude mice	10, 25, and 50 mg/kg	° Chrysin inhibits the tumor	[100]
Pancreatic cancer	MIA PaCa-2	25–100 µM	° Chrysin cleaved and activated caspase-9, ° Chrysin induced PARP cleavage	[102]
Liver cancer	HCC-LM3 xenograft model	30 mg/kg	° Chrysin suppressed tumor growth	[106]
	HepG2, Hep3B, Huh-7, HCC-LM3, Bel-7402 and SMMC-7721 cells	15–60 µM	° Chrysin inhibited HCC proliferation, Cleaved caspase-3	[106]
Breast cancer	MDA-MB-231 and MCF-7	100 µM	° In MDA-MB-231 cells, chrysin treatment decreased G0/G1 phases	[116]
	4T1 cells	60–100 µM	° Hypoxia-induced STAT3 tyrosine phosphorylation inhibited by chrysindent manner	[117]
	4T1 spontaneous metastasis model	100 and 250 mg/kg	° Chrysin suppresses growth of lung metastatic colonies	[117]
Cervix cancer	HeLa cells	10 and 15 µM	° Chrysin triggers cell cycle arrest ° Chrysin promotes apoptosis ° Chrysin modulates cell cycle regulatory genes expression	[120]
Endometrial cancer	HEC-1A and Ishikawa cells	0, 20, 40 and 80 µM	° Chrysin induced the apoptosis	[125]
Ovarian Cancer	OV90 and ES2	20, 50, and 100 µM	° Chrysin induced apoptosis	[126]
Prostate cancer	PC-3 cells	10 and 40 µM	° Cells treated with chrysin showed growth and induced apoptosis	[130]
Bladder cancer	T24 cells	20, 40, 80 µM	° Chrysin triggers the activation of caspase-3/9 and inhibits the STAT3 pathway	[131]
Renal cancer	N-nitrosodiethylamine induced renal carcinogenesis model	20, 40 mg/kg	° Tumor incidence by pretreated with chrysin	[133]
Thyroid cancer	HTH7 and KAT18 cells	25 and 50 mM	° Chrysin induces apoptosis	[135]
	HTh7 and KAT18	25 and 50 µM	° Chrysin treatment induced luciferase activity in cancer cells	[137]
	Subcutaneous Xenograft Tumor Model	75 mg/kg	° Chrysin Suppressed ATC tumor Growth	[137]
Lung cancer	A549	1–10 µM	° Chrysin activates AMPK in cultured lung cancer	[139]
Oral cancer	HSC4 and SCC25	100 and 200 µM	° Chrysin showed reduction in migratory ° Chrysin enhances apoptotic effect	[143]
Brain cancer	C6 glioma cells	30 and 50 mM	° Chrysin induces G1 phase cell cycle arrest	[147]
Skin cancer	375SM and A375P	0, 40, and 80 µM	° Chrysin-induced apoptosis ° Chrysin-induced autophagy	[148]
Leukemia		10 and 50 mg/kg	° Chrysin promoted macrophage phagocytosis ° Chrysin promoted NK cell activity of splenocytes	[150]

### 4.6. Anti-Diabetic Potential

The antidiabetic potential of medicinal plants is attributed to several mechanisms such as glucose regulation, the enhancement of insulin production, antioxidant activity, and anti-inflammatory effects. Moreover, natural compounds maintain the tissue architecture. The effects of chrysin on diabetic nephropathy were evaluated. Chrysin efficiently improved insulin resistance, obesity, renal function, as well as pathological injury in DN mice. Chrysin has been shown to enhance key indices and markers associated with lipid accumulation, oxidative stress, and inflammation, all of which play a role in the development or progression of diabetic nephropathy. Furthermore, chrysin positively impacted significant indicators related to lipid accumulation, as well as inflammation, contributing to the onset or progression of the condition [151]. The protective effects of chrysin against oxidative damage of streptozotocin (STZ)- induced diabetic rats were examined. The results showed significant increases in glucose, malondialdehyde (MDA), total cholesterol (TC), triglycerides (TG), and LDL-C, along with reductions in HDL-C, total protein, CAT, SOD, and GST in the untreated diabetic groups. However, these changes were improved in the CH-treated diabetic groups in a dose-dependent manner [152]. The antidiabetic potential of chrysin against streptozotocin (STZ)-induced diabetes was examined. It was reported that diabetic rats showed elevated levels of blood glucose, NO, as well as MDA in serum, along with reduced levels of pancreatic GLUT2, GSH, as well as insulin. Additionally, STZ injections resulted in increased serum levels of NF-κβ, TLR4, and HSP70, while CD4+ levels in serum decreased, with necrosis of pancreatic cells. These histological and biochemical changes were reversed in the glimepiride as well as chrysin groups [153].

### 4.7. Neuroprotective Effects

**Figure 6:**
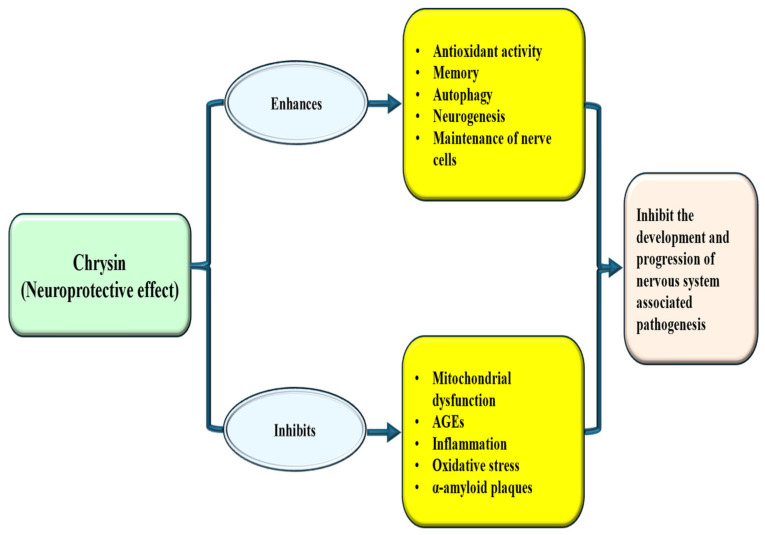
Neuroprotective mechanism of chrysin. Chrysin enhances antioxidant enzymes and neurogenesis and inhibits oxidative stress, inflammation and mitochondrial dysfunction. This ultimately inhibits/decrease the disease associated with central nervous system.

Natural compounds including chrysin have been extensively studied to assess neuroprotective potential through different mechanisms, such as reductions in oxidative stress and inflammation, and enhance cognitive function. Here, the roles of chrysin as neuroprotective and in different neurological disorders are discussed. This flavonoid showed role as neuroprotective [Table 4 and Figure 6]. The treatment of chrysin improves levels of GABA, monoamines, glutamic acid, and their metabolites in three brain regions, while also inhibiting DNA fragmentation markers like 8-HdG as well as BDNF. Additionally, it modifies the downregulation of Ca-ATPase induced by Clonazepam (CZP), a classic anti-anxiety drug treatment. Furthermore, Chrysin significantly reverses the behavioral changes observed which were raised by Y maze and open field tests changed by treatment of CZP [154]. The study sought to explore the potential protective effects of chrysin against memory impairments associated with hippocampal neurogenesis. The Methotrexate (MTX) group showed impairments in recognition and spatial memories. Furthermore, a decrease in neuronal cell survival, reduced cell division, and a decline in immature neurons were observed in the methotrexate group, factors that were not noticed in the groups that received both chrysin and MTX. These findings suggest that chrysin might enhance memory and alleviate neurogenesis deficits in rats treated with MTX [155]. In the experimental model of Parkinson’s disease, treatment with chrysin was found to decrease the loss of dopaminergic neurons [156]. Both free chrysin and CN-SLN were shown to reverse learning impairments and reduce neuroinflammation [157]. The neuroprotective properties of chrysin were checked. It was exhibited that chrysin reduced neurological deficit scores as well as infarct volumes. The increases in proinflammatory cytokine secretion and glial cell numbers caused by ischemia/reperfusion were meaningfully ameliorated by pretreatment of chrysin [158]. A study explored the effects of chrysin treatment in a Parkinson’s disease model, observing its protective impact on behavioral as well as cognitive changes, neuroinflammation and nitric oxide production [159].

Study result reported that the pre-treatment with chrysin protected degeneration of nigra-striatal neurons. Chrysin has been noticed to alleviate oxidative stress and improve motor dysfunction caused by MPTP. Furthermore, pre-treatment with chrysin prevented alterations in neurotrophic factors, inflammatory markers, and dopamine levels associated with MPTP exposure [160]. Another study confirmed the synergistic neuroprotective effects of chrysin, which enhanced the protective properties of protocatechuic acid. This combination led to increased cell viability and decreased lactate dehydrogenase release in 6-hydroxydopamine-treated PC12 cells. Additionally, the combination reduced dopaminergic neuron loss in both zebrafish as well as mice models [161]. Administration of Aβ25–35 (10 µg/rat) resulted in poor memory retention during behavioral tests. Treatment with chrysin ameliorated the memory deficits noticed in Aβ25–35-treated rats. Histopathological investigation of the hippocampus demonstrated noteworthy neuronal loss in rats given Aβ25–35, which was alleviated following chrysin administration [162].

### 4.8. Cardioprotective Effects

Natural compounds such as chrysin have been extensively researched for their cardioprotective potential, chiefly through mechanisms that reduce oxidative stress and inflammation. This discussion focuses on the role of chrysin as a cardioprotective agent and its effects on various cardio-associated pathogenesis [Table 4]. The cardioprotective role of chrysin against Cyclophosphamide (CP)-induced cardiotoxicity in rats was checked. The administration of CP significantly increased serum levels of cardiac injury markers and altered cardiac function. In contrast, coadministration of chrysin resulted in dose-dependent enhancements. Additionally, CP notably decreased the cardiac expression of regulatory T cell markers. Treatment of chrysin reversed these changes in a dose-dependent way. Histopathological investigation demonstrated that CP induced myocardial congestion, inflammatory cell infiltration, edema, and necrosis, which were progressively improved by chrysin [163]. A study found that cyclophosphamide (CyC) caused substantial cardiotoxicity, as demonstrated by clear increases in heart weight as well as cardiac function biomarkers. H&E-stained histopathological investigation revealed clear alterations in cardiac tissue. CyC also suggestively reduced RBC and WBC levels. However, treatment by chrysin effectively reversed these biochemical and histopathological changes [164]. The cardioprotective effects of chrysin were inspected. Administration of isoproterenol (ISO) meaningly increased the heart weight-to-body weight ratio, along with raising cardiac injury and inflammatory markers. Histopathological and ultrastructural analyses revealed damaging changes to the heart tissue. However, pre-treatment with 60 mg/kg of chrysin effectively reversed ISO-induced myocardial damage and prevented cardiac hypertrophy and fibrosis [165]. Chrysin treatment meaningfully reduced pulmonary vascular remodeling and improved collagen accumulation in the pulmonary artery and lung tissue. In vitro, chrysin notably reduced PASMC proliferation, collagen I expression, and collagen III expression, and the inhibitory potential of chrysin was accompanied by NOX4 expression inhibition, ROS production, and MDA generation [166].

### 4.9. Role in Reproductive System Associated Pathogenesis

The beneficial role of chrysin in the reproductive system of rats was examined. It was reported that chrysin meaningfully increased Glutathione (GSH), CuZn-SOD, CAT, and GSH-Px levels. Furthermore, sperm motility, serum testosterone levels, and sperm concentration suggestively increased, while the abnormal sperm rate significantly decreased with chrysin treatment [167]. The study assessed the impact of chrysin, a flavonoid found in Passiflora species, propolis, as well as honey, on female sex hormone levels and the histomorphology of the ovaries as well as uterus. When rats were administered by chrysin at 100 mg/kg, a prolonged estrous phase was observed. Moreover, the height of the luminal epithelium was increased in the chrysin-treated groups. Additionally, progesterone and luteinizing hormone (LH) levels were higher in chrysin-treated rats [168].

### 4.10. Role in Respiratory System Associated Pathogenesis

Chrysin has been confirmed to play an imperative role in inhibiting pathogenesis related to the respiratory system by various mechanisms [Table 4]. This flavonoid has established anti-inflammatory potential, which can help to decrease the respiratory system associated with disease complications. Chrysin alleviates ovalbumin (OVA)-induced airway hyperresponsiveness (AHR). Chrysin also decreases OVA-induced increases in the number of inflammatory cells. Furthermore, goblet cell hyperplasia, inflammatory cell infiltration, and the expression of α-SMA around bronchioles were decreased by chrysin [169].

The study investigated the protective role of chrysin against cadmium (Cd)-induced lung damage in rats. Findings showed that chrysin evidently reduced lung tissue MDA levels and suggestively enhanced antioxidant enzyme activities. Chrysin exerted anti-inflammatory potential in Cd-induced lung tissue. Moreover, chrysin up-regulated the Bcl-2 gene and reduced the side effects caused by Cd by modulating histopathological changes [170]. A study sought to evaluate the effectiveness of chrysin in countering pulmonary edema (PE) and pulmonary arterial hypertension induced by Alpha-naphthylthiourea (ANTU) in rats. Acute ANTU administration produced pronounced signs of pulmonary edema, reflected by elevated relative lung weight, greater lung fluid accumulation, increased pleural effusion volume, bronchoalveolar lavage fluid cell counts, and total protein levels. However, pretreatment with chrysin mitigated these ANTU-induced histological and biochemical changes [171]. The impact of chrysin on airway inflammation triggered by cigarette smoke in mice was examined. Exposure to cigarette smoke led to increased levels of inflammatory cytokines in bronchoalveolar lavage fluid (BALF) as well as higher MPO expression in lung tissue. In contrast, pretreatment with chrysin evidently suppressed the release of these cytokines, mitigated airway inflammation, and cigarette smoke-induced MPO expression decreased [30].

The anti-inflammatory role in a mouse model of Acute lung injury (ALI) induced by lipopolysaccharide (LPS) was investigated. In comparison to mice that were challenged with LPS alone, the treatment by chrysin led to a decrease in lung injury development, as demonstrated by histopathological results. Pre-treatment by chrysin also diminished inflammation and myeloperoxidase production in both the lung as well as BALF. Additionally, chrysin lessened lung edema by lowering vascular permeability in lung tissue [172].

### 4.11. Role in Digestive System Associated Pathogenesis

Chrysin has been shown to significantly contribute to inhibiting pathogenesis associated with the digestive system, mainly through reduction of inflammation [Table 4]. Chrysin displays prominent anti-inflammatory and antioxidant effects that alleviate complications associated with digestive system disorders. In this study, a dextran sulfate sodium–induced mouse model of ulcerative colitis was used to assess the therapeutic potential of chrysin. The findings showed that chrysin administration significantly reduced body weight loss and lowered disease activity index scores. Additionally, chrysin markedly reduced histological damage in the colon and decreased serum TNF-α levels [173]. A study result reported that chrysin pre-administration ameliorated inflammatory symptoms in mouse models of colitis and caused down-regulation of NF-*κ*B target genes in the colon mucosa [174]. The gastroprotective effects of chrysin were investigated in mouse models of gastric ulceration. In this study, it was found that chrysin (at a dosage of 10 mg/kg) effectively reduced macroscopic lesions and enhanced catalase activity in mice subjected to an absolute ethanol model. Additionally, it alleviated gastric ulcers induced by acetic acid by improving the expression of inflammatory genes, enhancing cell proliferation, and decreasing cellular apoptosis [175]. The protective action of chrysin was assessed in a model of indomethacin-induced gastric ulcer. Chrysin, at both 50 and 100 mg/kg doses, was found to enhance mucus secretion and effectively prevent increases in ulcer and lesion indices, acid secretion, and histological changes induced by indomethacin [176].

### 4.12. Anti-Arthritis Potential

The anti-arthritis potential of flavonoids has gathered considerable interest due to its anti-inflammatory and antioxidant potential that may help mitigate the symptoms of arthritis. Flavonoids reduce joint inflammation, leading to decreased pain and protects cartilage.

**Figure 7:**
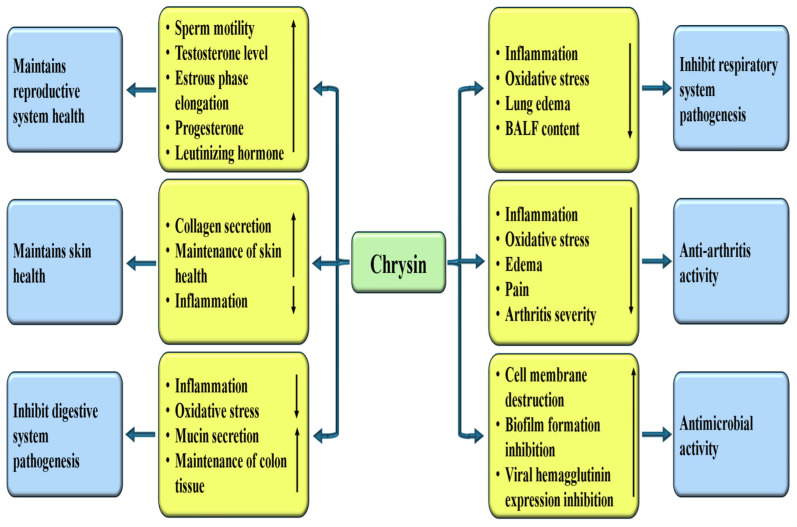
The prospective role of chrysin in influencing diverse disease pathogenesis. Chrysin enhances antioxidant enzymes and reduce oxidative stress and inflammation. This ultimately inhibits/decreases the spread of many diseases. The downward-pointing arrow shows downregulation, while the upward arrow denotes upregulation.

In this regard, chrysin as a flavonoid showed role as anti-arthritis through various mechanisms [Figure 7 and Table 4]. The study was planned to assess the anti-inflammatory as well as anti-arthritic activity of chrysin. Treatment with methotrexate (MTX), chrysin and their combination exhibited a prominent inhibition of paw oedema and pain, body weight restoration as well as immune organ weight as noticed by the ankle joints histology [177]. The study was made to assess the anti-arthritic activity of chrysin against complete. Chrysin treatment reduced the inflammatory cells, rheumatoid factor, arthritis score and erythrocyte sedimentation rate. Moreover, chrysin reduced infiltration of inflammatory cells, the severity of arthritis in joints, subcutaneous inflammation, bone erosion, cartilage erosion, and pannus formation [32]. The research aimed to investigate the anti-inflammatory effects of chrysin in knee osteoarthritis induced by monoiodoacetic acid. The findings designated that chrysin diminished synovial inflammation, lowered the secretion of pain-related substances, and increased both the paw withdrawal threshold as well as cold pain threshold in rats [178]. The influence of chrysin and its functional interaction with HMGB1 was investigated using a chondrocyte model of osteoarthritis. Findings demonstrated that chrysin suppressed chondrocyte apoptosis, decreased the expression of MMP13, collagenase, and IL-6 in a dose-dependent manner, and enhanced the expression of the collagen α1(II) chain in human osteoarthritic chondrocytes [179].

### 4.13. Role in Skin Health

Chrysin, with its antioxidant, anti-inflammatory, and photoprotective properties, is a significant contributor to skin health [Figure 7]. This compound acts as a shield, protecting the skin from oxidative stress caused by environmental factors. They also play a role in promoting collagen production, which is essential for skin elasticity. In a rat model of amiodarone extravasation–induced skin injury, chrysin treatment (20 and 40 mg/mL) meaningfully reduced the injury area as well as the levels of TNF-α and IL-6 on days 3, 7, and 10 compared with both the control group and the 10% DMSO solvent group [180]. Another study result reported that chrysin meaningfully improved imiquimod-induced disruption of skin barrier and skin lesions in mice [181]. The effects and underlying mechanisms of chrysin on photoaging and melanogenesis were examined. Chrysin enhanced collagen I secretion and confirmed anti-photoaging activity by reducing collagen I degradation, mitigating oxidative damage, and lowering the rate of HDF senescence. Moreover, chrysin showed inhibitory effects on melanogenesis and suppressing both melanin synthesis and the expression of melanogenic proteins [182]. A study was conducted to evaluate the protective effects of chrysin against UV-induced damage in HaCaT keratinocytes. The findings showed that chrysin reduced UVA- and UVB-induced ROS production, apoptosis, and COX-2 expression. In animal experiments, topical application of chrysin demonstrated effective percutaneous absorption and caused no skin irritation [183].

### 4.14. Anti-Microbial Properties

Chrysin, known for its antioxidant, anti-inflammatory, and antimicrobial properties, plays a vital role in combating pathogens. This compound acts as an antimicrobial by disrupting the cell membrane and inhibiting biofilm formation [Figure 7]. The study investigated the antibacterial effectiveness of chrysin when used alongside colistin against *Acinetobacter baumannii*. Results showed that both compounds worked synergistically, altering the bacterial membrane potential and damaging the external membrane of the bacteria. Furthermore, the combination of chrysin and colistin effectively reduced biofilm formation [184]. Another study assessed the in vitro antioxidant and antimicrobial properties of chrysin. The results revealed that chrysin exhibited significant scavenging activity and ferric-reducing antioxidant capacity. Additionally, chrysin effectively inhibited the growth of *Salmonella typhi* and *Bacillus subtilis*, with a minimum inhibitory concentration (MIC) of 1.25 mg/mL, comparable to that of chloramphenicol [185]. Anti-hepatitis B activity of chrysin was investigated. It was exhibited that chrysin decreases HBeAg, HBsAg secretion, supernatant HBV DNA as well as cccDNA, in a dose-dependent way [186].

**Table 4:** This flavonoid showed role in different pathogenesis through various mechanisms.

Activity	Study Type	Dose	Outcome of the Study	Refs.
Antidiabetic effects	Streptozotocin-induced diabetic rats’ model	20, 40, 80 mg/kg	° Chrysin treatment improves diabetes & its complications	[152]
	STZ-induced diabetes rat’s model	40 and 80 mg/kg	° Chrysin administration reduced blood glucose ° Chrysin elevated pancreatic GLUT2 content ° Chrysin caused reduction of NF-κβ	[153]
Neuroprotective effects	Clonazepam induced cognitive deficits rat model	50 mg/kg	° Chrysin treatment improves GABA, glutamic acid, monoamines, as well as their metabolites	[154]
	Parkinson’s disease mice model	10 mg/kg	° Chrysin attenuated the behavioral changes. ° Chrysin protects neuroinflammation	[159]
Cardioprotective effect	Cyclophosphamide-triggered cardiotoxicity rat model	25, 50, and 100 mg/kg	° Chrysin ameliorated impairment of cardiac contractility markers. ° Histopathological changes were ameliorated and downregulated iNOS expression by chrysin	[163]
	Cyclophosphamide-induced cardiotoxicity rat model	25 and 50 mg/kg	° Chy offered cardioprotective abilities via its antioxidant, and anti-inflammatory properties.	[164]
	Hypoxia-induced pulmonary hypertension rat model	50 or 100 mg/kg	° Chrysin treatment ameliorated cardiovascular remodeling and decreased collagen accumulation in pulmonary arteries	[166]
Role in respiratory system	Cadmium-induced pulmonary toxicity rat model	25 & 50 mg/kg	° Chrysin therapy reduces MDA levels in lung tissue and increases the activity of antioxidant enzymes levels ° Chrysin attenuated reduces histopathological changes	[170]
	ANTU-induced hypertension rat model	10, 20, and 40 mg/kg	° Chrysin inhibited pulmonary edema and pulmonary arterial hypertension	[171]
	Cigarette smoke-induced airway inflammation in mice model	10, 20 mg/kg	° Chrysin inhibits airway inflammation	[30]
	Lipopolysaccharide-induced lung injury mice model	10 & 25 mg/kg	° Chrysin ameliorated pulmonary edema and vascular leakage in mice	[172]
Role in digestive system	Chemically-induced colitis mouse model	25 mg/kg	° Chrysin administration attenuated colitis. ° Chrysin administration protect the colon crypt structures and reduces histologic inflammation.	[174]
Role in arthritis	CFA-induced arthritis rat model	25, 50, 100 mg/kg	° The combination treatment showed a prominent inhibition of paw oedema and pain and ameliorated arthritis	[177]
	Arthritis rat model	50 and 100 mg/kg	° Chrysin improved the histopathological parameters of arthritis ° Chrysin inhibited the degree of arthritis	[32]
	MIA-induced KOA rat model	10 mg/kg	° Chrysin reduced synovitis in vivo ° Chrysin alleviated Pain	[178]
Role in skin health	Skin injury rat model	10, 20 and 40 mg/mL	° Chrysin effective in reducing injury area, and reducing inflammation	[180]

## 5. Synergistic Effect of Chrysin and Potential Drug Interactions

**Figure 8:**
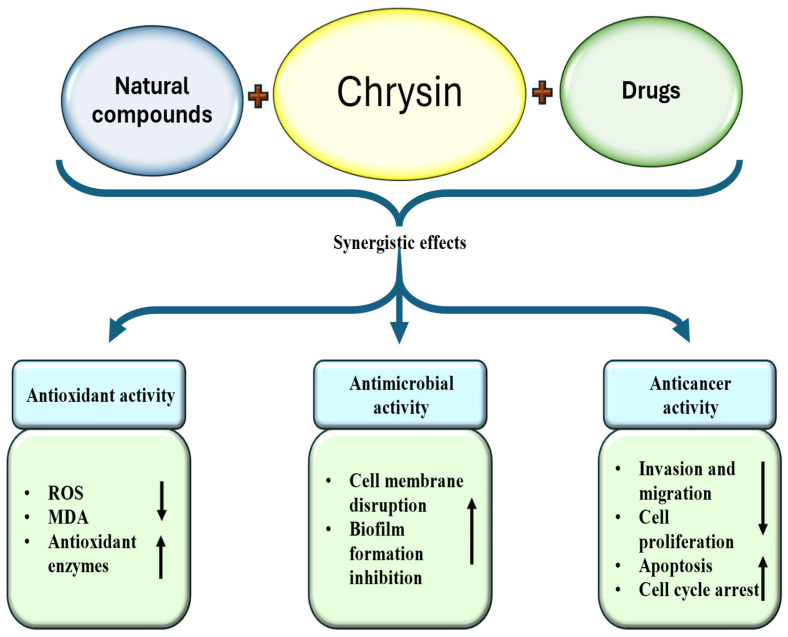
Synergistic effect chrysin. The downward-pointing arrow shows downregulation, while the upward arrow denotes upregulation.

Bioavailability is a substantial limitation of flavonoids, which can hinder their efficiency. However, when combined with other drugs, their activity can be improved. This synergistic approach can improve the absorption as well as efficacy of flavonoids, allowing them to manage pathogenesis. In this regard, the synergistic effect of chrysin denotes to the improved action when added to natural compound/drugs, leading to greater efficacies and enhanced bioavailability [Figure 8 and Table 5]. A study investigated the impact of chrysin and apigenin on colorectal cancer (CRC). The treatment with the combination of chrysin (25 µM) and apigenin (25 µM) meaningfully reduced cell clone numbers, migration, as well as invasion capability, whereas increased the cell apoptosis in both HCT-116 and SW480 CRC cell lines. The combination potential was higher than apigenin or chrysin alone. In the meantime, p-P38 and p-AKT downregulated by apigenin and chrysin treatment. The tumor inhibitive effect of apigenin combined with chrysin was clearly reversed by adding P38 agonist, anisomycin. chrysin (25 µM) combined with apigenin (25 µM) exhibited synergetic action in e growth and metastasis inhibition of CRC cells thru the activity of P38-MAPK/AKT pathway suppression [187]. The effects of chrysin and quercetin on MDA-MB-231 and MCF-7 breast cancer cells were studied. Notably, the combination of the two compounds enhanced their cytotoxic effects, especially when quercetin was preincubated. This combination led to cell cycle arrest in the sub-G0/G1 phase in MDA-MB-231 cells and modified the expression of caspases-3 and -8, ultimately promoting late apoptotic cell death. In summary, these results show that both compounds inhibited cell growth in a dose-dependent way, and the combination of quercetin enhanced the toxic effects of chrysin on the cell lines [116]. Another study confirmed that chrysin as well as colistin exert synergistic effects against *A. baumannii* by disrupting bacterial membrane potential and damaging the extracellular membrane. The combination also effectively inhibited biofilm formation. These findings suggest that chrysin–colistin combination therapy may represent a promising strategy for managing *A. baumannii* infections [184]. The combined potential of chrysin and kaempferol on ischemic rat brain was investigated. It was reported that infarct area, neurological score and NF-κB as well as STAT3 expression levels were meaningfully reduced. The evaluation of the neuroprotective synergistic effects of chrysin and kaempferol revealed therapeutic potential in mitigating cerebral ischemia by regulating the expression of pro-inflammatory mediators [188]. A recent study reported that treatment of MDA-MB-231 cells with chrysin in combination with radiation therapy (RT) caused synergistic antitumor properties. Chrysin synergistically potentiated RT-induced apoptosis in MDA-MB-231 as compared to monotherapies. Moreover, expression of Hypoxia-Inducible Factor 1-alpha (HIF-1α) was reduced in the cells exposed to combinational therapy [114].

In order to investigate a chemopreventive approach aimed at improving the effectiveness of breast cancer treatment, the combined antiproliferative effects of chrysin and silibinin were evaluated in T47D breast cancer cells. Cell viability assessments showed that both chrysin and silibinin, when used separately, reduced cell proliferation in a dose- and time-dependent way. Also, the combination of these two compounds caused in a synergistic activity that meaningfully enhanced growth inhibition in the breast cancer cell line. Moreover, combination drug synergistically down-regulated the mRNA levels of hTERT as well as cyclin D1 with the drugs used alone [189]. In a study on breast cancer, the combination of metformin and chrysin demonstrated pronounced synergistic cytotoxic effects on cancer cells. Moreover, this combination markedly downregulated the expression of cyclin D1 and hTERT genes in the T47D breast cancer cell line [118].

To enhance the therapeutic efficacy of 5-FU and overcome its resistance, the combined effects of chrysin and 5-FU were examined. The combination treatment significantly increased cytotoxicity compared with either chrysin or 5-FU alone. Mechanistically, the synergistic effect noted as induced S phase cell cycle arrest. These results indicate that chrysin and 5-FU exert anticancer effects through distinct pathways [190]. Chrysin alone or in combination with 5-FU reduced the number of aberrant crypt foci and the percentage of pathological lesions compared with azoxymethane. Moreover, the combination of chrysin with 5-FU resulted in a greater decrease in COX-2 [191]. Another experimentation reported that the combination of chrysin as well as cisplatin enhanced p53 phosphorylation and accumulation in HepG2 cells. Additionally, this combination promoted intrinsic apoptosis, increasing cytochrome c release and activating caspase-9, as well as activating caspase-8, respectively [192]. A study based on human glioma (U87) cancer cells reported that chrysin exhibited strong and dose-dependent antiproliferative activity. Additionally, when chrysin was combined with a low dose of cisplatin, the resulting growth inhibition was significantly enhanced. This combination not only led to increased induction of apoptosis but also caused more pronounced cell cycle arrest compared to treatment with chrysin or cisplatin alone. Furthermore, the combination of chrysin and cisplatin resulted in a higher percentage of in both early and late stages of apoptosis, and the combined treatment also affected the loss of mitochondrial membrane potential [193].

A study based on triple-negative breast cancer (TNBC) cell lines found that treating MDA-MB-231 cells with chrysin in combination with radiation therapy (RT) led to synergistic antitumor effects. The findings demonstrated that chrysin synergistically potentiated RT-induced apoptosis in MDA-MB-231 cells compared to the individual treatments of chrysin or RT alone. Moreover, exposure to the combination therapy resulted in reduced expression of HIF-1α in the cells. These results demonstrated the potential activity of chrysin as a radiosensitizer, demonstrating the synergistic anti-cancer effects of chrysin and RT in TNBC [114]. The effects of combined chrysin as well as kaempferol treatment were estimated in septic mice. Kaempferol alone increased the survival rate of CLP-challenged mice by up to 16%, while the combination of kaempferol and chrysin meaningfully doubled the 7-day survival rate, reaching 29%. The two compounds exhibited synergistic role by targeting numerous pathophysiological mechanisms involved in sepsis [194]. Another study demonstrated that the combination of pyrotinib and chrysin promotes autophagy in HER2-positive breast cancer via a unrecognized miR-16-5p/ZBTB16/G6PD axis [195].

Beyond its recognized pharmacological effects, preclinical studies indicate that chrysin and its metabolites may inhibit drug-metabolizing enzymes and influence drug transport mechanisms. The investigation focused on the inhibitory effects of certain flavonoids on the CYP3A4 enzyme. Among the flavonoids tested, chrysin demonstrated the strongest inhibitory effect on CYP3A4 activity. These results suggest that flavonoids hold the capacity to inhibit the CYP3A4 enzyme and interact with other drugs and medications [196]. The study examined the effects of chrysin, chrysin-7-glucuronide, and chrysin-7-sulfate on cytochrome P450 enzymes, as well as on organic anion-transporting polypeptides and ATP-binding cassette transporters. The findings indicated that chrysin conjugates serve as potent inhibitors of specific biotransformation enzymes, such as CYP2C9, as well as several transporters, including BCRP, OATP2B1, OATP1B1, and OATP1B3 [197].

Most studies are preclinical, conducted either in vitro or in vivo, providing preliminary evidence rather than definitive conclusions. The observed synergistic effects were identified using non-translatable dosages, which exceed physiologically achievable concentrations in humans, thereby limiting their clinical relevance. Furthermore, the limitation of safety, as well as toxicity data for chrysin when used in with other therapeutic agents.

**Table 5:** Synergistic effect with other compound/drugs in different pathogenesis.

Chrysin	**Compound/Drug**	**Activity/Role in**	**Study Type**	**Findings**	**Refs.**
	Apigenin	Colorectal cancer	In vitro	° The combination treatment migration, and invasion ability ° Apigenin combined with chrysin exhibited synergetic role in inhibiting the growth as well as metastasis	[187]
	Quercetin	Breast cancer	In vitro	° Quercetin as well as chrysin association induced cell cycle arrest	[116]
	Kaempferol	Neuroprotective	In vivo	° The kaempferol and chrysin combination alleviates cerebral ischemia	[188]
	Radiotherapy	Breast cancer	In vitro	° Chrysin synergistically potentiated apoptosis	[189]
	Silibinin	Breast Cancer	In vitro	° The synergistic antiproliferative effects was related to the down-regulation of cyclin D1 and hTERT genes	[190]
	Metformin	Breast Cancer	In vitro	° The combination of metformin as well as chrysin caused synergistic role in killing cancer cells ° Combination decreases in cyclin D1 and hTERT gene expression	[118]
	5-fluorouracil	Gastric Cancer	Invitro	° The combination treatment improved the anticancer effect	[190]
	5-FU	Colorectal Cancer	In vitro	° The combination of chrysin with 5-FU reduced COX-2 expression ° The combined chrysin boosted 5-FU efficiency	[191]
	Radiotherapy	Breast cancer	In vitro	° Treatment of breast cancer cells with chrysin in combination with RT showed synergistic antitumor activities	[114]
	Kaempferol	Sepsis	In vitro	° The combination treatment improved survival rate	[194]
	Pyrotinib	Breast cancer	In vitro	° The combined treatment enhances autophagy	[195]
	Colistin	Antimicrobial	In vitro	° The combination treatment displayed inhibitory potential on biofilm formation	[184]

## 6. Advances in Nanotechnology-Based Methods for Improving Chrysin Efficacy

**Figure 9:**
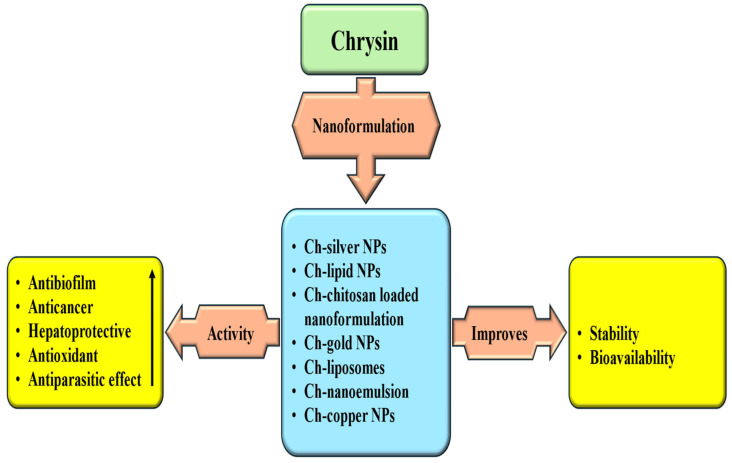
Nanoformulation based on chrysin.

Chrysin play an effective role in disease management through different mechanisms. Despite their valuable role, poor bioavailability limits the efficacies of this compound in disease management. Formulation of this compound using nanotechnology enhances its bioavailability and increases efficacy in disease management [Figure 9 and Table 6]. To increase the efficacies and bioavailability of chrysin, various approaches have been utilized and its effects in pathogenesis checked.

Nanoformulation increases the activity, stability and bioavailability of chrysin. The study designed to measure the antibiofilm effects of chrysin-fabricated silver nanoparticles (nano-chrysin) against *Pseudomonas aeruginosa*. Nano-chrysin inhibited biofilm produced by *P. aeruginosa* PAO1, showing MIC values between 50 and 3.13 µg/mL, demonstrating greater potency than chrysin or silver nanoparticles alone [198]. Another study examined the role of chrysin as an efficient adjuvant along with nanostructured lipid carriers (NLCs) to enhance the cytotoxicity of doxorubicin (Dox) in MCF-7 breast cancer cells. The average size of the nanoparticles was 105 ± 2 nm, which was established by SEM. The findings designated that treating the cells with chrysin-loaded nanostructured lipid carriers (NLCs) increased the apoptosis rate from 21.11 ± 5.72% to 27 ± 3.13%. Additionally, the proportion of cancer cells in the sub-G1 phase rose to 12 ± 2.1% in comparison to the untreated cells. Moreover, mRNA expression levels of Nrf2, HO1, NQO1, as well as MRP1 showed a notable reduction when compared to the control group. The findings suggest that the delivery of chrysin along with NLCs could improve the efficacy of Dox by providing inhibitory effects on drug efflux pumps and drug detoxification enzymes [199].

The anti-biofilm activity of chrysin-Loaded chitosan nanoparticles against *Staphylococcus aureus* was investigated. At sub-Minimum Inhibitory Concentration (sub-MIC), the nanoparticles showed increased anti-biofilm effectiveness against *S. aureus* as compared to its bulk counterparts, chitosan and chrysin. The reduction in cell surface hydrophobicity and the production of exopolysaccharides confirmed that the nanoparticles had an inhibitory effect on the early stages of biofilm formation. Additionally, growth curve analysis showed that at sub-MIC, the nanoparticles did not kill *S. aureus* [200]. Chrysin conjugated gold nanoparticles were constructed and checked for their anti-leishmanial action. The nanoparticles had an average size of 20 nm and achieved a drug loading efficiency of 90%. The IC_50_ value for AuNPs-CHY was 0.8 µg/mL, which was meaningfully more than CHY (2.19 µg/mL). Gold nanoparticles, which are already recognized for their anti-leishmanial properties, along with conjugated chrysin, showed a reduced parasite burden in mammalian macrophages. This finding displayed that this biofunctionalized nanoformulation might be used as a possible therapeutic tool against leishmaniasis [201]. The liposomal chrysin (LC) was prepared and examined the effect and potential mechanism on hepatic ischaemia–reperfusion (HIR). The average particle size of the LC was measured at 129 ± 13.53 nm, with a polydispersity index of 0.265 ± 0.021 and a zeta potential of −34.46 ± 4.14 mV. The encapsulation efficiency and drug loading were found to be 95.03 ± 2.17% and 16.4 ± 0.8%, respectively. Following the administration of LC, the concentration of chrysin in plasma increased by 2.54 times, while in liver tissue, it rose by 1.45 times. It was demonstrated that LC pre-treatment decreased HIR-induced liver injury as well as inhibited cell apoptosis. Furthermore, LC pre-treatment decreased malondialdehyde and reactive oxygen species levels and suppressed the inflammatory response, as demonstrated by reduced neutrophil infiltration as well as lower IL-6 and TNF-α levels. Additionally, LC pre-treatment noticeably inhibited NLRP3 activation, confirmed by decreased expression of cleaved caspase-3, NLRP3, ASC, cleaved caspase-1, and IL-1β [202].

The study designed to make a more bioavailable form of chrysin and to investigate its effects on neuropathy. To improve its bioavailability, chrysin was formulated with PEGylated liposomes. A series of experiments were conducted to evaluate the antidiabetic effects of the chrysin-loaded liposomes (Chr-PLs) and, by extension, their potential to improve Diabetic neuropathy (DN). According to the results, the prepared Chr-PLs exhibited an average particle size of approximately 134 nm. They displayed even distribution of particle sizes. The maximum entrapment efficiency of 90.48 ± 7.75% was achieved. Chr-PLs effectively decreased blood glucose levels by 67.7% and elevated serum acetylcholinesterase levels by 40% compared to Diabetic neuropathy rats. Moreover, Chr-PLs reduced the expression of ER stress-related genes. They were found to upregulate the expression levels of miR-301a-5p while simultaneously downregulating other related miRNAs. Additionally, formulation enhanced the expression of autophagic markers in the sciatic nerve. Histopathological investigation revealed that Chr-PLs effectively inhibited degeneration of the sciatic nerve [203].

Chrysin-loaded bilosomes were prepared and estimated for their potential to enhance the hepatoprotective effects of the drug. The optimized chrysin-loaded bilosomes exhibited a spherical morphology with an average particle size of 232.97 ± 23 nm. They had a polydispersity index of 0.35 ± 0.01 and a zeta potential of −44.5 ± 1.27 mV. The entrapment efficiency was 96.77 ± 0.18%, while the drug loading percentage was 6.46 ± 0.01. Additionally, the release efficiency after 48 h was found to be 42.25 ± 1.04. Chrysin-loaded bilosomes demonstrated superior antioxidant activity compared to free chrysin. This was consistent with histopathological results, which showed substantial improvement in serum hepatic biomarkers in CCl_4_-intoxicated mice treated with chrysin-loaded bilosomes versus those treated with free chrysin [204].

Another study assessed the efficiency of chrysin-loaded nanoemulsion (CH NE) against epilepsy in rats. The formula optimized showed a droplet size of 48.09 ± 0.83 nm, PDI 0.25 ± 0.011, sustained release, and good stability. The chrysin treatment resulted in a reduction of seizure scores and improved both behavioral and histological changes. Additionally, chrysin induced the polarization of microglia from the M1 to M2 type, which helps to reduce inflammation. The CH NE formulation was shown to significantly enhance drug delivery to the hippocampus in rats compared to the CH suspension [205]. Another study designed to assess copper nanoparticles (CuNPs) synthesized using chrysin in mice bearing Ehrlich solid tumor. CuNPs were characterized with irregular round sharp shape with size range of 21.19–70.79 nm and plasmon absorption at 273 nm. EC mice exposed to combined treatment of CuNPs as well as radiation exhibited a noticeable decrease in tumor volume, ALT and CAT, calcium, and creatinine GSH. Comparing histopathological outcomes of treatment groups ends that combined treatment was of better efficiency, indicating tumor tissue regression and increase in apoptotic cells [206]. The role of chrysin-loaded PCL-PEG-PCL nanoparticles were estimated in a breast cancer cell line. Real-time PCR results showed that encapsulated chrysin employed a stronger antitumor effect on the expression of FTO, hTERT, and BRCA1 genes compared to free chrysin [207].

Although preclinical studies have shown that chrysin and its various nanoformulations improve solubility, bioavailability, and therapeutic effectiveness both in vitro and in animal models, the lack of clinical studies remains a significant limitation. As a result, the direct application of these findings to human health outcomes remains limited. Few studies directly compare different platforms in terms of stability, biodistribution, toxicity, and therapeutic efficiency The need of well-structured clinical studies to assess the safety, pharmacokinetics, optimal dosing, and efficacy of chrysin in humans. Bridging this gap is crucial to determine whether the benefits observed in preclinical research can be safely and effectively translated to clinical practice.

**Table 6:** Chrysin based nanoformulation and role in disease management.

Formulations	Activity	Study Types	Outcomes	Refs.
Chrysin Fabricated Silver Nanoparticles	Antibiofilm		° Biofilm produced by *P. aeruginosa* PAO1 was found to be inhibited by nano-chrysin	[198]
Chrysin loaded nanostructured lipid carriers	Anti-cancer		° Chrysin-loaded NLCs had synergistic effects on cellular uptake of Dox.	[199]
Chrysin-Loaded Chitosan Nanoparticles	Antibiofilm Activity		° The nanoparticles showed improved anti-biofilm efficiency	[200]
Chrysin-conjugated gold nanoparticles	Anti-leishmanial		° Chrysin-conjugated gold nanoparticles neutralize Leishmania parasites with high efficacy	[201]
Liposomal chrysin	Prevent liver injury	In vivo	° LC pre-treatment reduced liver injury	[202]
Chrysin-loaded PEGylated liposomes	Protection of diabetic neuropathy	In vivo	° Chr-PLs decreased blood glucose levels and elevated serum acetylcholinesterase levels	[203]
Chrysin loaded bilosomes	Hepatoprotective activity	In vivo	° It was noted as improvement in serum hepatic biomarkers of mice treated with chrysin loaded bilosomes	[204]
Chrysin-loaded nanoemulsion	Role in epilepsy	In vivo	° The formulation improves drug delivery	[205]
Chrysin Encapsulated Copper Nanoparticles	Anti-tumor	In vivo	° formulation showed a reduction in tumor volume	[206]
Chrysin-loaded PCL-PEG-PCL nanoparticle	Antitumor effect		° The encapsulated chrysin have higher antitumor effect	[207]

## 7. Safety and Toxicity of Chrysin

Understanding the toxicity and safe dosage of any drug is vital before its use. Toxicity and safety evaluations are important for establishing safe dosage levels, understanding potential side effects, and ensuring patient safety. Research in preclinical models has shown that chrysin is relatively safe. The suggested daily dosage of chrysin is 0.5–3 g [21,208,209]. While individuals typically consume small amounts of flavonoids through their daily diet, higher doses can potentially lead to toxicity [208,209]. The study aimed to assess the safety of chrysin by evaluating its toxicity following acute and subchronic oral administration in rats. In the AOT assessment, an oral dose of chrysin at 5000 mg/kg resulted in a 40% mortality rate. In the sub-chronic toxicity analysis, daily administration of chrysin at 1000 mg/kg significantly reduced body weight, while liver weight increased notably in male rats. Additionally, significant alterations in blood parameters were observed in the chrysin-treated group. Notably, there was a marked increase in hepatic and renal oxidative-nitrosative stress among rats receiving chrysin at 1000 mg/kg. However, no significant changes were detected in electrocardiographic, hemodynamic measurements, left ventricular function, or lung function tests. Histological examination revealed renal and hepatic abnormalities in rats treated with 1000 mg/kg of chrysin [210].

## 8. Clinical Study Based on Chrysin

Chrysin has demonstrated a multifaceted role in the management of various pathological conditions through several mechanisms, including antioxidant, anti-inflammatory, neuroprotective, hepatoprotective, and cardioprotective effects, as evidenced by both in vitro and in vivo studies. However, the body of clinical trial research on chrysin across various diseases remains limited. Further investigation is warranted to elucidate its therapeutic potential in clinical settings. Some studies based on humans are discussed here. The study was made to determine whether a daily treatment over 21 days with propolis and honey, which includes chrysin, would affect urinary testosterone levels in male volunteers. Chrysin is known for its ability to inhibit aromatase, potentially preventing the conversion of androgens to estrogens, with a resultant increase of testosterone, finally measurable in urine samples. However, the results indicate that there were no substantial changes in testosterone levels among the volunteers after 7, 14, or 21 days of treatment, compared with baseline values and with the control subjects measured simultaneously [211]. A study was conducted to investigate the safety of combining single-agent irinotecan (CPT-11) with chrysin. Twenty patients with previously treated advanced colorectal cancer received chrysin twice daily for one week before and after treatment with single-agent CPT-11. Loperamide use, as well as bowel frequency and consistency, was monitored throughout the study. Results showed no significant toxicities attributable to chrysin. The incidence and severity of delayed diarrhea were mild, with only 10% of patients reporting grade 3 toxicity [212]. Oromuco-adhesive films designed for the buccal delivery of Propolis extract (PPE) encapsulated in niosomes were developed to address oral recurrent aphthous ulcers (RAU). The resulting films containing niosomal PPE were assessed for their swelling properties, mucoadhesion, as well as elasticity. A total of 24 patients with recurrent aphthous ulcers (RAU) were evenly split into two groups: one receiving the medication and the other receiving a placebo. The study aimed to evaluate the onset of ulcer size reduction, pain relief and complete healing. The clinical findings indicated that the oromuco-adhesive films remained adhered for 2 to 4 h in both groups. In the medicated group, a reduction in ulcer size was observed by the second or third day, with complete healing occurring within the first 10 days of treatment. Additionally, pain relief lasted for over 4 to 5 h as compared to the placebo group [213].

A study was conducted to evaluate the efficacy of a nutritional supplement aimed at boosting serum testosterone levels while also inhibiting the conversion of ingested androgens into dihydrotestosterone and estrogens in healthy men aged 30 to 59. Participants were randomly assigned to take either DION (which contained 625 mg of chrysin, 300 mg of androstenedione, 540 mg of saw palmetto, 150 mg of dehydroepiandrosterone, 300 mg of indole-3-carbinol, and 750 mg of Tribulus terrestris each day) or a placebo for 28 days. DION led to an increase in serum levels of androstenedione, dihydrotestosterone, free testosterone, and estradiol. However, it also resulted in a decrease in serum HDL-C levels. While the combination of androstenedione with herbal products raised serum free testosterone levels in older men, these herbal supplements did not stop the conversion of androstenedione into estradiol and dihydrotestosterone [214]. A study on the oral disposition of the flavonoid chrysin was conducted with healthy volunteers. Seven participants received 400 mg of chrysin. The concentrations of chrysin and its metabolites were measured in plasma, urine, and feces. Peak plasma concentrations of chrysin ranged from 3 to 16 ng/mL, while levels of plasma chrysin sulfate were found to be 30 times higher. In urine, chrysin and chrysin glucuronide were detected in amounts ranging from 0.2 to 3.1 mg and 2 to 26 mg, respectively. The majority of the administered dose was excreted in feces as chrysin [21]. A study was conducted to evaluate the effectiveness of an androgenic nutritional supplement aimed at increasing serum testosterone levels while also inhibiting the formation of dihydrotestosterone and estrogen in healthy men aged 30 to 58. Participants were randomly assigned to take a nutritional supplement (AND-HB) daily, containing 300 mg of androstenediol, 300 mg of chrysin, 480 mg of saw palmetto, 1500 mg of gamma-linolenic acid, 450 mg of indole-3-carbinol, and 1350 mg of Tribulus terrestris. There were no significant differences in basal serum total testosterone, prostate-specific antigen, and estradiol concentrations between the different age groups. However, basal serum-free testosterone levels were higher in the 30-year-olds compared to the 50-year-olds. Additionally, basal serum dihydrotestosterone (DHT) and androstenedione concentrations were significantly greater in the 30-year-olds than in those aged 40 or 50. Consuming AND-HB led to elevated levels of serum androstenedione, free testosterone, DHT, and estradiol over the 4-week period. These findings suggest that taking androstenediol in combination with herbal products does not inhibit the formation of estradiol and dihydrotestosterone [215].

## 9. Conclusions, Limitations and Future Direction

Flavonoids are important secondary metabolites found in fruits, vegetables, and tea; they are widely associated with reduced disease risk due to their antioxidant and anti-inflammatory properties. Among this group, chrysin has gained significant attention in health management for its ability to modulate various biological activities. Chrysin effectively scavenges free radicals and reduces oxidative stress, a key factor in cardiovascular and neurodegenerative diseases, as well as other chronic conditions. As an anticancer agent, chrysin modulates numerous cell signaling pathways, including the regulation of inflammation, angiogenesis, the PI3K/AKT pathway, and the induction of apoptosis, autophagy, and cell cycle arrest. These mechanisms highlight its potential role in cancer prevention and therapy. However, despite its promising therapeutic profile, chrysin faces substantial restrictions. The most critical challenge is its low bioavailability, driven by poor solubility, limited permeability, and rapid metabolism. These pharmacokinetic disadvantages restrict its clinical application and decrease the likelihood of achieving therapeutic concentrations in humans. While preclinical studies have shown that chrysin and its various nanoformulations improve bioavailability, solubility, and therapeutic efficacy in both in vitro and animal models, the lack of clinical studies remains a significant challenge. Therefore, the direct application of these findings to human health outcomes is still limited. Most available data are based on preclinical studies, and well-designed clinical trials are urgently needed to validate chrysin’s efficacy and safety in humans. Concerns regarding toxicity and interactions with other drugs are still poorly understood. Additionally, many animal studies utilize high doses that may not be safe for human use. Addressing these gaps will require extensive research efforts, including the development of advanced formulations, mechanistic studies, and clinical trials. Strengthening these areas is essential to fully uncover chrysin’s therapeutic potential and facilitate its successful translation into clinical use.

## Data Availability

No new data were created or analyzed in this study. Data sharing is not applicable to this article.

## Abbreviation

Abbreviation	Full form
IL	Interleukin
TGF-β1	Transforming Growth Factor-beta
NF-κB	Nuclear factor kappa-light-chain-enhancer of activated B cells
LDH	Lactate dehydrogenase
TNF	Tumour Necrosis Factor
iNOS	Inducible Nitric Oxide Synthase
ROS	Reactive Oxygen Species
RNS	Reactive Nitrogen Species
CDDP	Cisplatin
CCL_4_	Carbon tetrachloride
COX-2	Cyclooxygenase-2
BUN	Blood Urea Nitrogen
5-FU	5-Fluorouracil
DSS	Dextran sodium sulfate
MMP	Matrix Metalloproteinase
Egr-1	Early growth response-1
HCC	Hepatocellular carcinoma
VEGF	Vascular endothelial growth factor
Fe-NTA	Ferric nitrilotriacetate
LDL	Low-density lipoprotein
CZP	Clonazepam
ISO	Isoproterenol
CAT	Catalase
GSH	Glutathione
GSH-Px	Glutathione Peroxidase
OVA	Ovalbumin
BALF	Bronchoalveolar lavage fluid
LPS	Lipopolysaccharide
HIF-1α	Hypoxia-Inducible Factor 1-alpha
CHNE	Chrysin-loaded nanoemulsion
OSCC	Oral squamous cell carcinoma
MAPK	Mitogen-activated protein kinas
PE	Pulmonary edema
ANTU	Alpha-naphthylthiourea
